# Mechanical adaptivity of red blood cell flickering to extrinsic membrane stiffening by the solid-like biosurfactant β*-*Aescin

**DOI:** 10.1016/j.bpj.2025.03.027

**Published:** 2025-04-01

**Authors:** Lara H. Moleiro, Diego Herráez-Aguilar, Guillermo Solís-Fernández, Niccolo Caselli, Carina Dargel, Verónica I. Dodero, José M. Bautista, Thomas Hellweg, Francisco Monroy

**Affiliations:** 1 Department of Physical Chemistry, Complutense University of Madrid, Madrid, Spain; 2 Translational Biophysics, Health Research Institute (imas12), Hospital 12 de Octubre, Madrid, Spain; 3 Physikalische und Biophysikalische Chemie, Universität Bielefeld, Bielefeld, Germany; 4 Faculty of Experimental Sciences, Francisco de Vitoria University (UFV), Madrid, Spain; 5 Molecular Imaging and Photonics, Department of Chemistry, KU Leuven, Heverlee, Belgium; 6 Department of Biochemistry and Molecular Biology, Faculty of Veterinary, Complutense University of Madrid, Madrid, Spain; 7 Translational Malaria Laboratory, Health Research Institute (imas12), Hospital 12 de Octubre, Madrid, Spain

## Abstract

β*-*Aescin is a natural additive employed for treatments of vascular insufficiency, hence its impact in red blood cell (RBC) adaptivity has been conjectured. Here, we report a study about the mechanical impact of the membrane stiffener aescin on the flickering motions of live RBCs maintained at the homeostatic status. An active flickering, or nonequilibrium fluctuation dynamics has been revealed by mapping flickering motions in single RBCs treated or not with aescin. Experiments show that active RBC flickers adapt mechanically to β-escin, unlike the passive thermal fluctuations observed in lipid bilayers without an active skeleton. Mechanical connections for active flickering are theoretically argued to exist between an effective viscoelastic softness bestowed by the spectrin membrane cytoskeleton and the observed stiffness imposed by aescin as a rigidity modulator. From the unveiled diffusive mechanics, we model an adaptive RBC homeostasis that recapitulates the active flickering phenomenon as an optimal membrane softness upon a regulated friction as observed under aescin-induced membrane hardening. From a physiological perspective, RBC flicker adaptiveness to rigidization is discussed according to regulatory principles of energy conservation and minimal dissipation.

## Significance

*Red blood cell (RBC) flickering*, or the dance of RBC life, is observed under an optical microscope as the highly deformable erythrocytes exhibit dynamic shape changes at adaptation to context. RBCs undergo large shape deformations as mechanically stressed with the biosurfactant β-Aescin, a natural stiffening agent. RBC membrane flickering has been known since the early 1950s, and it was first analyzed by Brochard and Lennon in their seminal 1975 paper. The modulation of transmitted light is due to shape deformations, leading to a fluctuation dynamics observable at focusing on the cell equator. Flickering can be quantified in relation to RBC mechanics and is believed to serve biological functions, such as controlling resistance to flow and preventing cell adhesion. In capillary microcirculation, for instance, RBC flickering involves squeezing deformations that enable navigation through extremely narrow capillaries. Under certain physiological or pathological conditions, the shape and movements of RBCs may become altered in response to the circulatory context, with Aescin emerging as a flickering modulator with pharmacological relevance.

## Introduction

Red blood cells (RBCs), or erythrocytes, are the most abundant cell type in the human body, playing a crucial role in transporting respiratory gases during their approximately 120-day lifespan (1,2). In circulation, RBCs enucleate as flexible discocytes with a high membrane/cytoplasm ratio, facilitating cell deformability and gas exchange (3,4,5,6). Traditionally seen as mere respiratory carriers, RBCs are also considered immunological adaptors, binding molecules and pathogens in the circulatory system (7,8,9). Homeostatic hemodynamics arises from RBCs’ adaptability to pathophysiological conditions in the bloodstream (10,11,12). This deformability relies on specialized mechanobiology enabling a robust and efficient membrane remodeling (13,14,15). The adaptable erythroid membrane has two asymmetrically intercoupled structural elements: 1) the outer heterogeneous lipid bilayer composed of phospholipids (including phosphatidylinositol), sphingomyelin, and cholesterol, providing a fluid support for protein functioning (16), and 2) an underlying cytoskeletal meshwork made of flexible spectrin filaments, structured internally by ankyrin, protein band 3 and 4.1, short actin filaments, and other actin-associated proteins, including tropomyosin motors (15,17). Mechanically, erythroid deformability involves passive resistance to lateral stretching, low shear rigidity, and active bending softness varied by cytoskeletal flexions (18,19,20). From a molecular mechanistic wisdom, the reconfigurable membrane mosaic imparts functional fluidness under physiological adaptivity (18,19,20). Hence, nonadaptive changes can impair RBC membrane deformability, leading to disease (21,22).

As circulating RBCs squeeze through capillaries, their deformable membranes spontaneously remodel, allowing them to recover their normal discocyte shape (13,23,24). This dynamic ability, known as flickering, is a hallmark of healthy RBCs (25,26,27,28,29). In our study, we captured microscopy images of a living RBC undergoing flickering fluctuations around the discocyte shape when mechanically stressed by a stiffening agent (see Video S1). Active RBC motions are facilitated by ATP-dependent membrane rheology, which restores flexible membrane properties (30,31,32), likely due to the active spectrin skeleton (33,34), regulated by force deformations driven by active membrane proteins (35). Metabolic out-of-equilibrium states maintain these active flickering fluctuations in living RBCs (30,31,32,33), enabling them to adapt their deformability to environmental changes (36,37). Unlike nonliving systems with a passive deformability undergone as near-Gaussian fluctuations at mechanical equilibrium, living RBC flickers exhibit active mechanical properties (38,39,40,41). Hence, the living RBCs indefectibly undergo mechanical irreversibility observable as breakdown of the fluctuation-dissipation theorem (42), often interpreted as high effective temperature due to nonequilibrium activity (39,40,43). Recent studies show that nonequilibrium (active) RBC flickering results in irreversible dissipation of internal energy to surroundings (44), being externally controllable by optical devices such as holographic tweezers (45). Therefore, RBC flickering is nowadays envisaged as an active deformability process dominated by heterogeneous (anomalous) diffusivity (39,40,41,42,43,44,45), being inherently homeostatic since regulating adaptive nonequilibrium states. Flickering adaptiveness reflects the physiological ability of living RBCs to dynamically adjust their heterogeneous membrane properties, ensuring cell deformability and survival in response to mechanical changes (1,5,9).

Video S1. Visualizing dynamic fluctuations of a human erythrocyte using phase-contrast microscopy

In this work, we explore the mechanical adaptability of human RBCs to the natural saponin β-Aescin (also known as escin), a membrane stiffener known for its antiedematous, vasoconstrictive, and anti-inflammatory properties (46). Escin is a hydrophobic, water-soluble biosurfactant with high adsorption activity on lipid membranes (47,48), forming compact complexes with membrane cholesterol (49,50). Akin to endogenous cholesterol, β-Aescin exhibits dual mechanics on model membranes providing flexural stiffening followed by lateral fluidization (51). Structural analyses have demonstrated a compacting integration of adsorptive β-escin into model lipid bilayers (48,50,51), e.g., the zwitterionic phospholipid dimyristoylphosphatidylcholine (DMPC), which is fluid at physiological temperature (52,53). Previous studies performed in vitro with living RBCs indicated that escin causes fatal hemolysis above 4 mM, a much higher concentration than clinical oral doses (∼10–40 mg/kg/day, corresponding to 50 *μ*M maximum in plasma (54), also higher than the critical micellar concentration (55)). Further in vivo studies with endothelial cells suggest that escin impacts cholesterol homeostasis, enhancing vascular protection (46). This therapeutic effect is linked to the integrity of the actin cortex under cholesterol organization (56). Those findings with living RBCs suggest that treatment with exogenous escin affects mechanical cell homeostasis, specifically their bare membrane flexibility dynamically regulated by endogenous cholesterol (18,57,58,59). Understanding escin-adaptive interactions with the erythroid membrane is of significant medical interest (60), although its physicochemical effects on RBC flickering have not yet been analyzed. Using life-imaging microscopy performed in living RBCs at the single-cell level (29,40,45), we have revealed the mechanical adaptability of the erythroid membrane to added escin, leading to homeostatic adjustments that affect flickering activity within escin-tolerability limits. We have also studied DMPC bilayers as control models to test escin-driven membrane stiffening under passive deformability. In our experiments, we exploit high-performance digital methods for rapid segmentation of flickering contours imaged by ultrafast videomicroscopy (29). Analyzing the flickering time series, we infer energy landscapes for actively adaptive processes. Based on the evidence raised, we depict the theoretical basis to describe active flickering adaptability as a nonconservative mechanical field established within a statistical mechanics framework. From experimental results, we postulate that RBC flickering is physiologically regulated to maintain functional deformability and mechanical adaptability under homeostasis.

## Materials and methods

### Chemicals and buffers

β-Aescin was purchased from Sigma (pharmaceutical primary standard), used without further purification (48,49,50,51). 1,2-Dimyristoyl-*sn*-glycero-3-phosphatidylcholine (DMPC) and 1,2-dioleoyl-*sn*-glycero-3-phosphoethanolamine-*N*-(lissamine rhodamine B sulfonyl) (RhPE) was supplied by Avanti Polar Lipids. DMPC was dissolved in chloroform/methanol mixture (1 mg/mL final concentration). All the other chemicals were obtained for Merck-Sigma. Ultrapure water was obtained from a Milli-Q system (Millipore; resistivity higher than 18 MΩ cm; organic residual lower than 1 ppb). We prepared enriched phosphate buffer (PBS+) complemented with glucose (130 mM NaCl, 20 mM Na_3_PO_4_, 10 mM glucose, and 1 mg/mL bovine serum albumin). This PBS+ buffer induces the production of ATP through glycolysis.

### Preparation of RBCs: Morphotypes

Human RBCs were freshly collected by venipuncture from healthy donors. Blood aliquots (20 *μ*L) were suspended in PBS+ (250 *μ*L) and minimally processed by centrifugation and rinsing three times (10 min at 5000 × g). The RBC pellet was resuspended in PBS+ (1:15, v/v) with varying escin concentrations (0–40 mM). Samples (10 *μ*L) were transferred to a microscopy chamber (37°C) and sealed with a cover glass to prevent evaporation. For immunochemistry fixation, RBCs were incubated in PBS with 4% paraformaldehyde for 12 h to induce protein cross-linking and membrane rigidization. RBCs were classified based on their shape using standards from the International Council for Standardization in Hematology (ICSH). We identified four main RBC types: healthy discocytes (circular and elliptical), echinocytes, and fuzzy spherocytes (ghosts). Other abnormal shapes were considered debris and excluded from the cell count (not exceeding 1%).

### Giant unilamellar vesicles

Lipid giant unilamellar vesicles (GUVs) made of DMPC were prepared using the electroformation method as optimized by Mathivet et al. (61). Briefly, lipid powder was dissolved in a chloroform/methanol mixture (2:1, v/v) at 1 mg/mL. RhPE was added for fluorescence detection (1 *μ*g/mL; final concentration <0.1%, w/w, with respect to lipid). A 20–30 *μ*L drop of the solution was deposited on an ITO slide, and the solvent was evaporated under a dry nitrogen stream. The ITO chambers were hydrated with 200 mM sucrose solution and placed in an oven at 38°C, above the DMPC melting temperature (23.6°C). The chamber was then connected to an AC generator (8 Hz, 1.8 V) for 3 h to induce electroswelling. To enhance GUV sedimentation and visualization, samples in 200 mM sucrose were diluted in a slightly higher concentration glucose solution (208 mM). For escin adsorption, DMPC vesicles were exposed to a 40 *μ*M escin solution.

### Hemoglobin absorbance: Hemolysis essay

Absorbance measurements were performed using an Agilent 8453 UV-vis spectrophotometer (Agilent Technologies, Rattingen, Germany) with quartz cuvettes (Hellma, Mülheim, Germany). A 25 *μ*L aliquot of RBC suspension was diluted in 150 *μ*L of PBS+ containing varying concentrations of escin (see preparation of RBCs: morphotypes). For the positive control, RBCs were resuspended in water to induce hypotonic shock. PBS+ buffer without RBCs served as the negative control. Samples were incubated for 1 h, then centrifuged for 5 min at 5000 × g. The supernatant containing hemolyzed cell content was transferred to the spectrophotometer, and absorbance was recorded at 340, 420, 542, and 577 nm, where free hemoglobin has distinct absorption peaks. The negative control provided a near-zero baseline, indicating that hemoglobin remained within the untreated RBCs.

### Confocal fluorescence microscopy

Confocal laser scanning microscopy images were acquired using a Nikon AX inverted confocal microscope, controlled by the Nikon Imaging Suite. The microscope was equipped with a Nikon Ti2-E body, an AX scan head unit, and a CFI Plan Apochromat Lambda D 100× oil immersion objective. For membrane staining, RBCs were incubated with wheat germ agglutinin (WGA) linked to Atto-647 (Thermo, cat no. W32466) at a 1:500 dilution from the original 1 mg/mL stock. The RBCs were incubated with the diluted WGA-647 in PBS for 30 min at room temperature, then washed twice with PBS. After fluorescence labeling, the RBCs were mounted onto glass slide coverslips and imaged using confocal laser scanning microscopy.

### RBC morphotyping: Circularity analysis

Living RBCs were observed in fresh samples using a phase-contrast microscope (TE2000, Nikon). Cytometric morphotyping images were captured at 100× magnification (1.45NA, Plan APO oil immersion objective) using an ORCA camera (32-bit, 2048 × 2048 resolution, Hamamatsu), with a lateral resolution of 0.12 *μ*m in the x,y-plane. An average of 10 cells were analyzed per field (minimal of 50 fields analyzed, typically 500 cells per case). The equatorial membrane rim was detected using the same contour algorithm applied in flickering fluctuation analysis (62) (see life fluctuation imaging), adapted for high-throughput segmentation of thousands of cells per field. Image processing, contour detection, and data analysis were performed using Mathematica 13.3 (Wolfram Research). For contour segmentation, images were denoised using a Gaussian filter (2 px) and binarized with Otsu’s variance maximization method. Morphological classification was based on discocyte circularity thresholds (based on measuring principal curvature $R_{1}$ and $R_{2}$, orthogonal): *discocytes*, equatorially circular red cells with a mean radius ($\bar{R}$), measured within 1% tolerance according to the mean sample size, and a canonical circularity ($c=R_{1}/R_{2}>0.95$); *elliptocytes*, cells with lower circularity than normal discocytes ($c=R_{1}/R_{2}\leq 0.95$); *echinocytes,* further distilled by relative contourness, $\Delta =\left(D-2\pi \bar{R}\right)/2\pi \bar{R}>0.1$ (where D is the segmented contour length); *erythroid ghosts*, all spheroidal objects lacking membrane contour contrast. Morphometric analysis was restricted to suspended cells with minimal morphological alterations.

### Life fluctuation imaging

To track cell contour flickering (62), we used high-velocity videomicroscopy combined with subpixel segmentation for precise detection of movements over different timescales (29,40,59). We employed an inverted bright-field microscope (TE2000, Nikon) with an ultrafast CMOS camera (Photron FastCam Mini AX100, 540 kfps max rate, 1 Mpixel, 32 GB RAM). Instantaneous deformations were measured along the equatorial membrane contour as ${\delta h}_{i}\left(l_{i},t\right)=R_{i}\left(t\right)-\bar{R}$ (where $l_{i}$ is the cell’s rim coordinate at each membrane emplacement and $\bar{R}$ the mean radius). Each equatorial RBC contour was segmented into N membrane emplacements of lateral size L ($=2\pi \bar{R}/N$) that constitute altogether the flickering ensemble (subindex i varies from 1→N=2048). Drift corrections for translation and rotation were applied relative to the cell’s barycenter (29). Local flickering amplitudes were determined as due to membrane softness calculated from deformation variances, i.e., $\sigma_{i}^{2}\left(l_{i}\right)=\frac{1}{n}\left\langle {\delta h}_{i}^{2}\left(l_{i};t\right)\right\rangle$ (the bracket ⟨⟩ indicates time-averaging over the time series obtained from a given flickering sequence of length $n=10^{5}$). The ensemble-averaged flickering variances are defined over all the microstates explored along the whole membrane contour in each single cell, i.e., $\Sigma^{2}\equiv \frac{1}{N}\sum_{i}^{N}\sigma_{i}^{2}\left(l_{i}\right)$. Good statistics were achieved for time averaging performed over long time intervals (typically, we record time series ΔT=5s longer, sampled at 2 kHz frame rate Δt=0.5ms; thus, the number of explored microstates corresponds to $n=\frac{\Delta T}{\Delta t}\geq 10^{4}$ frames). Ensemble averages were performed over RBC populations in each healthy state, either nontreated or treated with escin (M≥20 cells). Experiments with living RBCs (and DMPC-GUVs) were performed at controlled temperature T=37±0.5ºC (well above the melting temperature of the structural lipid; $T>T_{m}\approx 23.6ºC$ for DMPC). At low temperature ($T<T_{m}$), the living RBCs undergo basal flickering (the solid lipid GUVs do not fluctuate anymore). Our approach (62) surpasses conventional methods that use slower videomicroscopy and lower performance image analysis (63). Advantages of our method include its nonintrusive nature, high velocity, and deep segmentation, ensuring unbiased measurements of membrane fluctuation dynamics (29,59).

### Stochastic mechanics

At the single-cell level we detected the instantaneous flickering displacements ${\delta h}_{i}\left(l_{i},t\right)$, along with the experimental flickering series (t) and the membrane rim coordinate ($l_{i}$). These deformations correspond to the possible microstates explored at localized membrane emplacements segmented from the equatorial cell contour, with elements of lateral size $L=2\pi \bar{R}/N\approx 25\;\text{nm}$. The flickering ensemble consists of the fluctuating collective of possible deformation microstates $\delta h_{i}$ (for i=1 to N=2048), diffusively explored by all membrane emplacements consistent with the macroscopic characteristics of each cell population. We monitored single-cell contour deformations over time, providing a detailed mapping of erythrocyte membrane dynamics as a stochastic process (29,40).

The local displacements ${\delta h}_{i}$ were considered small elastic deformations relative to the undeformed bounding membrane. For mechanical analysis, we used the conceptual framework of stochastic mechanics in elasticity bounding fields (64). Hence, we define the energy landscape as a near-harmonic potential $U_{i}\left({\delta h}_{i}\right)\approx \frac{1}{2}G_{eff}^{\left(i\right)}{\delta h}_{i}^{2}$, characterized by an effective elasticity modulus $G_{eff}^{\left(i\right)}$, which recapitulates the surface modes of membrane elasticity (e.g., lateral stretching and transverse flexion) (65). In the limit of frictional overdamping, the stochastic equation for the flickering motions is given in the familiar Langevin form (35):

(1) $$\zeta_{i}v_{i}+f_{i}\left({\delta h}_{i}\right)=\xi_{i}\left(t\right)$$

The first term is the frictional drag under flickering velocity $v_{i}$ (the friction coefficient $\zeta_{i}=6\pi L\eta_{i}$ as due to the local membrane viscosity $\eta_{i}$). The second term is the restoring force due to effective membrane elasticity, i.e., the local flickering force here invoked:

(2) $$f_{i}=f_{pass}^{\left(i\right)}+p_{on}f_{act}^{\left(i\right)}=-\partial U_{i}/\partial {\delta h}_{i}\approx -G_{eff}^{\left(i\right)}{\delta h}_{i}\text{,}$$

being $G_{eff}^{\left(i\right)}\approx G_{0}-p_{on}\Delta G_{i}$ an effective local rigidity, where $G_{0}$ is the passive rigidity of the membrane, $p_{on}$ the probability of the flickering element to become activated, and $-\Delta G_{i}$ the corresponding active softening.

The stochastic forces $\xi_{i}\left(t\right)$ consist of passive thermal noise (uncorrelated) and active components (correlated kicking forces), driving the flickering modes of deformation under direct (propulsive) forces causing membrane softness (35); the active direct component is given by:

(3) $$p_{on}f_{act}^{\left(i\right)}\equiv \Delta G\Lambda_{0}\Leftrightarrow \pm f_{0}$$

where $\Lambda_{0}$ represents kicking displacements.

Within the linear elasticity harmonic approximation, the membrane’s flexural responsivity comprises large-scale tension modes governed by facilitated surface tension (γ) and small-scale curvature modes governed by the cytoskeleton’s bending rigidity (κ). In the weak-coupling limit, the passive restoring force is approached by (66):

(4) $$-f_{pass}^{\left(0\right)}\approx \gamma \partial_{l}{\delta h}_{i}+\kappa \partial_{ll}{\delta h}_{i}\approx G_{0}{\delta h}_{i}$$

where γ is the lateral tension, and $\kappa \equiv G_{0}d^{3}$ the bending modulus, defined for an apparent membrane thickness d, which is specified as an effective parameter significantly larger than the bilayer thickness (28,65). An effective quasi-two-dimensional plate geometry is assumed to describe membrane dynamics (67). Partial derivatives refer to the lateral membrane coordinate (l).

### Flickering power and diffusivity

The dynamical interplay between membrane viscoelasticity and deformation forces driving flickering processes was analyzed in reciprocal frequency space ($\omega ↔t^{-1}$) (68). Spectral analysis was performed by fast Fourier transform (29). The local mechanical response was obtained from flickering displacements under power spectral density defined as (29,68):

(5) $$PSD_{i}\left(\omega ,q\right)\equiv \frac{k_{B}T}{\omega {\tilde{G}}_{eff}\left(\omega \right)}=\frac{1}{L}\int \int \delta h_{i}^{2}\left(l_{i},t\right)e^{i\omega t}e^{iql}dl\;dt$$

which represents the flickering energy involving deformation modes in a membrane element of lateral size L, and comparable effective height d (67).

In Fourier space, passive modes span along the fluctuation series at wave vectors q with a dispersion relation $\omega ∼q^{\alpha }$ (i.e., $\alpha_{\gamma }=2$, for tension modes, and $\alpha_{\kappa }=4$ for bending) (28,65). Thus, the natural timescale of membrane motion is given by a diffusive frequency defined as a viscoelastic ratio across surface tension and bending rigidity regimes (33,65,67):

(6) $$\omega \left(q\right)=\frac{\gamma_{i}q+\kappa_{i}q^{3}}{4\eta_{i}}\approx \frac{G_{eff}^{\left(q\right)}}{\eta_{i}}$$

which determines passive diffusive relaxation due to friction in a membrane element of size L, on the same order of the apparent membrane thickness (i.e., L≈d≈50−100nm). The diffusive frequency $\omega_{D}\approx \gamma^{3/2}/\eta \kappa^{1/2}$ determines the crossover between surface tension and bending rigidity at mechanical balance against viscous friction. Further q-mode integration provides the local thermal contribution as a bimodal spectrum arising from passive tension and bending modes, such as

(7) $${PSD_{pass}\left(\omega ;G_{eff}\right)\equiv \left\langle \delta h_{i}^{2}\left(\omega \right)\right\rangle }_{q}=\int_{0}^{\infty }\delta h_{i}^{2}\left(\omega ,q\right)dq\Leftrightarrow \frac{k_{B}T}{{\omega G}_{eff}^{\left(i\right)}}$$

calculated upon linear intrinsic elasticity at equilibrium $G_{eff}^{\left(i\right)}\left(\omega ;\gamma_{i},\kappa_{i}\right)\approx \left(4\gamma_{i}/R\right)+12\pi \left(d/R\right){\left(2\eta_{i}^{2}\kappa_{i}\right)}^{1/3}\omega^{2/3}$, where the first term is the local Laplace pressure and the second one accounts for the bending stress (39,69,70).

### Active flickering: Fractional diffusivity

Nonequilibrium (active) membrane fluctuations (35), driven by cytoskeletal protein forces, generate enhanced motion against surface tension or bending (30,31,32,33,34,35,36,37,38,39,40). Following Gov’s model of nonthermal membrane fluctuations (35), active cytoskeletal proteins are assumed to induce direct kicking forces ($\pm f_{0}$), within an intrinsic rate for force generation ($\omega_{A}$). These active flickering fluctuations emerge as a nonequilibrium contribution due to absolute kicking forces ($∼f_{0}^{2}$), with strength inversely proportional to the surrounding microviscosity (η); for direct kicking forces (35):

(8a) $${PSD}_{act}^{\left(D\right)}\left(\omega \right)\approx p_{on}{\left(\frac{f_{0}}{4\eta }\right)}^{2}\frac{\omega_{A}}{\omega^{2}\left(\omega_{A}^{2}+\omega^{2}\right)}$$

while for curvature-induced motion (35):

(8b) $${PSD}_{act}^{\left(C\right)}\left(\omega \right)\approx p_{on}{\left(\frac{f_{0}R^{2}}{4\eta }\right)}^{2}\frac{\omega_{A}}{\omega^{2/3}{\left(\kappa /4\eta \right)}^{4/3}\left(\omega_{A}^{2}+\omega^{2}\right)}$$

In general, at low flickering frequencies ($\omega <\omega_{A}$), an effective behavior is expected:

(8c) $${PSD}_{act}^{\left(f\right)}\left(\omega \right)\propto p_{on}{\left(\frac{f_{0}}{4\eta }\right)}^{2}{\left(\omega /\omega_{A}\right)}^{-2\alpha }$$

Under the fractional exponent α, pure active diffusivity—dominated by direct kicking forces (at $\omega \approx \omega_{A}\ll \omega_{D}$) —corresponds to α=1 (surface tension regime). In contrast, curvature-dominated forces (at $\omega \gg \omega_{D}$), lead to confined anomalous diffusivity, resulting in α<1 (bending correlations); in the pure-bending regime, α=1/3 (70). Since the active and passive components are, in principle, uncorrelated (35,43), the ensemble-averaged flickering spectrum can be rewritten in a bicomponent form as:

(9) $${\bar{PSD}}_{flick}={\bar{PSD}}_{pass}\left(\omega /\omega_{D};G_{eff}\right)+\phi \left(p_{on}\right){PSD}_{act}^{\left(f\right)}\left(\omega /\omega_{A};f_{0},\alpha \right)$$

where ϕ represents the average fraction of active elements.

### Spectral analysis: Rheology

In experimental terms, the average spectrum, $\bar{PSD}\equiv \frac{1}{N}\sum_{i}{PSD}_{i}$, represents the mean power dissipated by the flickering ensemble across its membrane emplacements (N=2048). Local fluctuations were assumed to be diffusively overdamped, with mobility rates defined from the statistical displacements as $v_{i}\left(t\right)\equiv \lim_{\delta t\to \Delta t}\frac{\delta h_{i}}{\delta t}=\frac{1}{\Delta t}{\left\langle {\left[h_{i}\left(t+\Delta t\right)-h_{i}\left(t\right)\right]}^{2}\right\rangle }^{1/2}\Rightarrow \sqrt{\frac{2D_{i}}{\Delta t}}$ (calculated for sampling times Δt). We examined apparently diffusive kinetics using the mobility autocorrelation function based on the generalized Green-Kubo theorem (71); for the phenomenological diffusivity, we considered $D_{i}\left(l_{i}\right)=\int \left\langle v_{i}\left(t\right)v_{i}\left(t+t^{\prime }\right)\right\rangle dt^{\prime \;}$(71). In Fourier space, the complex diffusivity and spectral density are related through a generalized friction coefficient ${\tilde{\zeta }}_{i}\equiv \left(6\pi L/\omega \right){\tilde{G}}_{eff}\left(\omega \right)$, incorporating viscoelastic memory described by the complex modulus ${\tilde{G}}_{eff}\left(\omega \right)=G_{i}\left(\omega \right)+i\omega \eta_{i}\left(\omega \right)$ . Therefore, we define the diffusive memory function related to the power spectral density (PSD) as follows (68):

(10) $${\tilde{D}}_{i}\left(\omega \right)\equiv \frac{k_{B}T}{{\tilde{\zeta }}_{i}\left(\omega \right)}=\int v_{i}\left(t\right)v_{i}\left(t+t^{\prime }\right)e^{i\omega t}dt↔\omega^{2}{PSD}_{i}\left(\omega \right)$$

Although membrane diffusivity is typically understood as a microscopic measure of the mobility of molecular species within the membrane, in our rheological context, we define membrane diffusion as the flickering mobility, which is expressed within the mesoscopic stochasticity framework above defined.

## Results

We conducted experiments on RBC flickering, a key indicator of membrane deformability and mechanical adaptability in homeostasis. We first assessed RBC viability under the mechanical stress induced by β-escin, followed by a biophysical analysis using live imaging.

### Toxicity evaluation of β-escin in human RBCs

**Figure 1:**
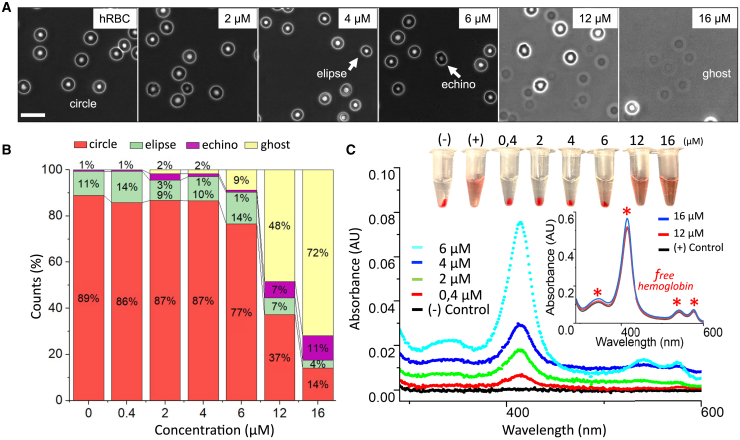
Morphotype RBC count as morphological changes and hemolytic effects of β-escin on erythrocytes. (*A*) Sequence of images of erythrocytes treated with escalating concentrations of β-escin (0, 2, 4, 6, 12, and 16 *μ*M), showing a progressive transformation of cell morphology and the appearance of ghost cells (ruptured erythrocytes) at higher concentrations. Scale bar, 10 *μ*m; field size: 60 × 60 *μ*m^2^. Erythrocytes are classified into four categories based on their morphology: healthy cells (both circular and elliptical forms), diseased cells (echinocytes), and ruptured erythrocytes (ghosts). (*B*) Bar chart representing the percentage distribution of each cell type based on analysis of 100 images like those shown in (*A*) for each β-escin concentration (*N* = 10^3^ cells typically, counted across almost 100 cytology images similar to those portrayed in Fig. 2*A* for each concentration). (*C*) UV-vis absorbance spectra of cell supernatants following treatment with various concentrations of β-escin. The emergence and intensification of characteristic hemoglobin absorption peaks (at approximately 340, 420, 542, and 577 nm) mean increasing hemolysis. Inset: magnified view of absorbance curves at high β-escin concentrations and positive control (cells lysed by osmotic shock using distilled water), where all characteristic peaks are clearly visible. The upper photograph shows the Eppendorf tubes corresponding to each concentration analyzed. The settling of intact RBCs at the bottom and free hemoglobin in the supernatant is observable.

To investigate the cytotoxic effects of β-escin, we studied cell viability in live RBCs using morphological cytometry and UV-vis spectroscopy measurements of hemolysis. The data in Fig. 1 illustrate these functional assessments.

#### Morphological changes induced by β-escin

Fig. 1*A* shows a typical morphological examination of healthy RBCs, referred to as the morphotype cell count. RBC morphotypes were determined across increasing doses of β-escin, from zero (control group of untreated cells, mostly discocytes) to 2–16 *μ*M of soluble β-escin. Fig. 1 *B* quantifies the morphological transition from normal to altered, with bar charts showing the percentage distribution of each cell type, including healthy cells (both circular and elliptical forms), diseased cells (echinocytes), and ruptured erythrocytes (ghosts). Up to a concentration of 4 *μ*M, there were no significant changes in the normal discocyte morphotype. However, at 6 *μ*M, there was a noticeable decrease in normal discocytes, indicating the onset of β-escin-induced morphological changes, i.e., toxicity. Thus, we established the RBC dysfunctional threshold at 4 *μ*M escin. Above this critical dose, RBCs primarily transitioned to the spherical shape of ghosts, indicating cell membrane rupture and release of cellular contents. We determined β-escin to be a hemolytic agent at doses above 12 *μ*M.

#### Hemoglobin release under treatment with β-escin

Fig. 1*C* displays UV-vis results for the hemolytic effect of β-escin on RBCs treated with varying escin concentrations. At lower β-escin concentrations, considered nonhemolytic based on cytometric analysis (Fig. 1 *B*), a slight peak appeared around 420 nm, indicating that, even at low concentrations (∼0.2 *μ*M), β-escin began to stress the cells. Cytotoxic stress was confirmed at concentrations around 4–6 *μ*M, where characteristic peaks for free hemoglobin were observed, indicating hemolytic release of hemoglobin into the solution (54). To complement the spectroscopic data, we also imaged samples at each β-escin concentration (Fig. 1 *C*, *photographs on top*). A progressive appearance of free hemoglobin was evident in the supernatant, as indicated by the color change from white to red, correlated with a decrease in RBC sediment. Cytometric analyses confirmed RBC viability up to β-escin concentrations of 4–6 *μ*M, with full hemolysis occurring at doses above 12 *μ*M.

### Mechanical adaptivity of flickering deformations to β-escin: Single-cell membrane fluctuation imaging

**Figure 2:**
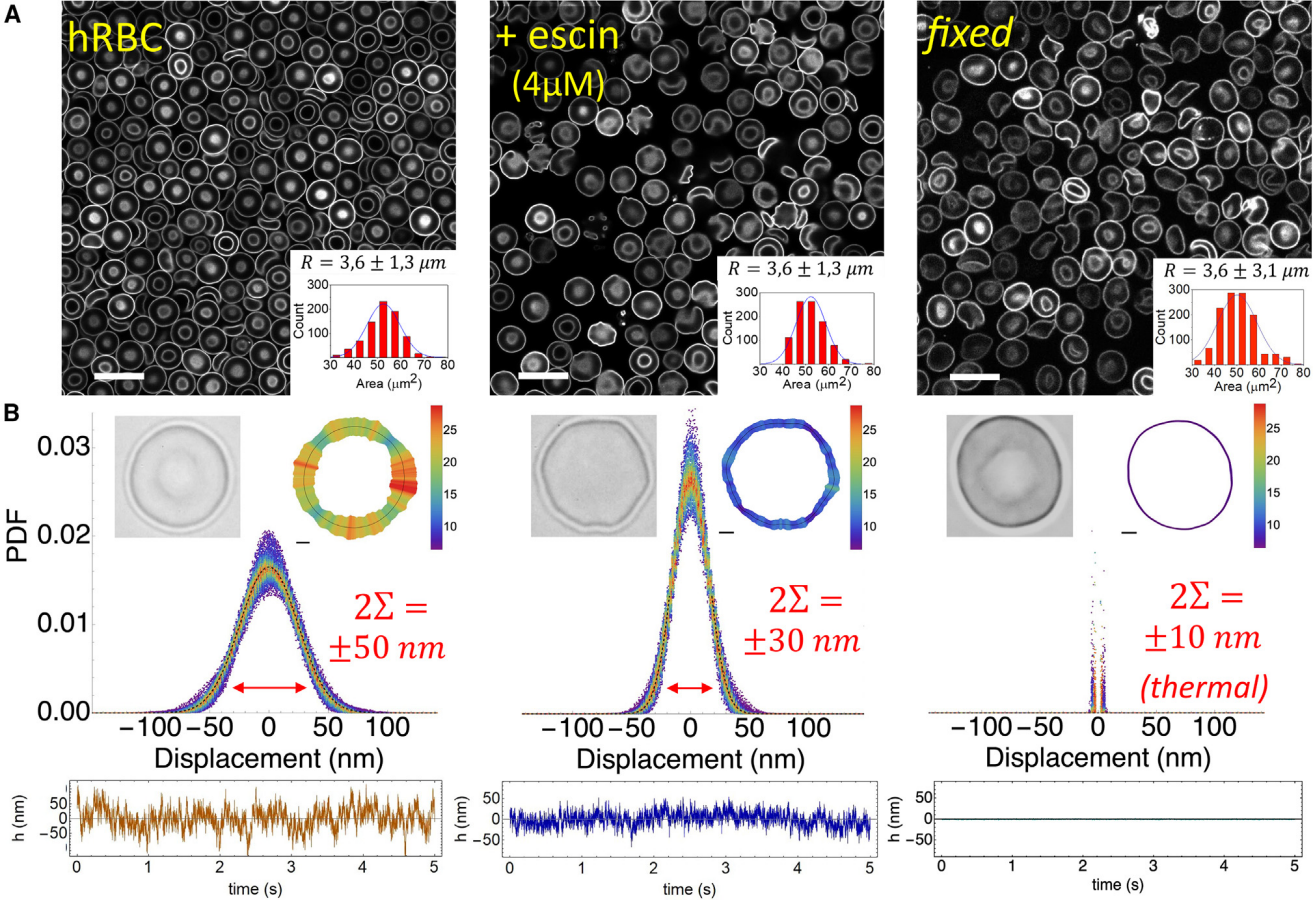
Flickering motions in living red blood cells treated with β-escin. In-depth evaluation of the erythrocyte membrane dynamics in RBCs under escin treatment at subtoxic concentrations (c≤4mM). (*A*) Population flickering deformations revealed by confocal live imaging in erythrocyte samples representative of the normal discocyte morphotype (*left*: nontreated cells; *right*: treated cells). (*B*) Single-cell spatial mapping of the flickering deformations, or local temperatures measured in normal discocytes displayed as representative specimens. In the uppermost images we showcase photographs for single erythrocytes (phase contrast microscopy; *left*), and corresponding spatial maps of flickering “temperatures” calculated to represent the local strength of the membrane deformations (flickering amplitudes plotted as standard deviations σ; *right*). The local effective temperatures are assumed proportional to the flickering variance $T_{eff}\;∼\;\sigma^{2}$ (see “temperature” scale defined for fluctuating amplitudes). The mapping colors indicate local effective temperatures proportional to local displacements (*red* corresponds to high $T_{eff}\gg T$, and *blue* to low [bath] temperature T). The central panels show experimental data for the probability density functions (PDFs) of the squared displacements accumulated over the whole RBC flickering ensemble (Σ is the standard deviation of the mean flickering motions around the discocyte shape). The majority of deformation events span within two PDF standard deviations (defining 2Σ as the characteristic displacements under 95% probability). For untreated cells they appear quite broad indicating kicking activity ($\Lambda_{0}\equiv 2\Sigma_{act}\approx \pm \;50\;\text{nm}$; *left*). However, for escin-treated RBCs they appear meaningfully narrower and peaked around zero indicating passivation ($2\Sigma_{pass}\approx \pm 30\;\text{nm}$; *right*). Typical time series are also shown showing flickering as stationary motions around the bounding membrane (*bottom panels*).

Fig. 2 provides insight into the flickering behavior of living RBCs as captured by live imaging. Fig. 2 *A* shows different flickering shapes in RBC populations: untreated (hRBC; *left panels*), treated with a high (nontoxic) dose of β-escin (hRBC + escin; *middle panels*), and fixed with protein cross-linking (fRBC; *right panels*). Cytometric analysis showed no significant changes in cell size within the population’s intrinsic variability ($\bar{R}=3.6\pm 1.3\;\mu m$ SD; see histogram insets). Live imaging revealed heterogeneous flickering deformations in both the collective RBC population (Fig. 2 *A*) and single RBCs (Fig. 2 *B*). These deformations appeared as symmetric membrane changes normally distributed around the equatorial cell radius (see also Video S1). A detailed analysis of adaptable RBC flickering activity is provided in the discussion, comparing local energy deformability between treated and untreated cells.

#### Flickering deformations: Probability density function

In Fig. 2 *B*, deformation amplitudes are represented as a heatmap on an arbitrary scale of local effective “temperature,” which reflects either the local kicking activity level or its equivalent interpretation as membrane softness. Both are proportional to the squared deformation, given by $\sigma_{i}^{2}\left(l_{i};p_{on}\right)\equiv N^{-1}\sum_{i=1}^{N}{\delta h}_{i}^{2}\left(l_{i}\right)\propto k_{B}T_{eff}^{\left(i\right)}$. This variance is calculated over all membrane locations (N), and is primarily influenced by $p_{on}$, the probability of the flickering element being activated as a membrane kicker capable of locally softening the membrane (35,39,40,42,43). The flickering amplitudes were found by averaging the local deformations over time, often exceeding the size of individual flickers, which can push the membrane over significant distances when activated, i.e., $\sigma_{i}\left(l_{i};p_{on}=1\right)\approx \left|\Lambda_{0}\right|≳L$ (see RBC morphotyping: circularity analysis and life fluctuation imaging). The analysis reveals highly diverse spatial patterns indicating two types of membrane flickering zones based on their mechanical softness (Fig. 2 *B*, *top panels*): 1) scattered “hot spots” showing significant deformation variances, indicating high local activity ($\phi \equiv N_{m}/N\approx 20-40\%$ of fully activated membrane sites ($p_{on}\approx 1$), thus ${\delta h}_{hot}^{2}\approx \Lambda_{0}^{2}\gg \sigma_{i}^{2}$; colored in *red*); 2) numerous “colder” areas displaying shorter displacements, suggesting lower activity ($1-\phi \equiv \left(N-N_{m}\right)/N\approx 40-60\%$ of typically passive sites ($p_{on}=0$), fluctuating thus within the standard deviation ${\delta h}_{cold}^{2}\leq \sigma_{i}^{2}$; colored in *blue*); 3) the remaining sites exhibit intermediate activity with a lower probability of activation than in hot spots (intermittent; $0\ll p_{on}<1$). The statistical distributions of the flickering deformations are represented by probability density functions, or PDFs (Fig. 2 *B*, *central panels*). They follow a near-Gaussian form $PDF\left\{{\delta h}_{i}\left(l_{i}\right)\right\}\propto e^{-{\delta h}_{i}^{2}/2\sigma_{i}^{2}}$ compatible with a normal distribution of deformability microstates. These flickering fluctuations, observed as stochastic movements around the discocyte shape, appear stationary and stably bounded to the membrane, even in the hottest emplacements (Fig. 2 *B*, *bottom panels*). Hence, the Boltzmann distribution gives the probability that the RBC adopted a given deformation ($\delta h_{i}$) i.e.,

(11) $$PDF\left\{{\delta h}_{i}\left(l_{i}\right)\right\}\propto \exp\left[-\frac{U_{i}\left(\delta h_{i}\right)}{k_{B}T}\right]$$

This assumes a near-harmonic energy $U_{i}\left(\delta h_{i}\right)\approx \frac{1}{2}G_{eff}^{\left(i\right)}{\delta h}_{i}^{2}$, under local apparent softness causing the flickering variance $\sigma_{i}^{2}\left(p_{on}\right)\equiv k_{B}T/G_{eff}^{\left(i\right)}$, upon environmental temperature (T). To extract statistically significant information, the mean variance was calculated as an ensemble average $\Sigma^{2}\equiv N^{-1}\sum_{i}^{N}\sigma_{i}^{2}\left(l_{i}\right)$, where the summation extends over the N-contour emplacements (Fig. 2 *B*, *central panels*) (64).

#### Flickering variances and kicking activity: Membrane softness

Assuming internal equilibrium in each RBC discocyte and biological variability within a population, the flickering ensemble should replicate under different conditions (i.e., healthy untreated, treated with escin, or completely passivated; see Fig. 2, *central panels*). For healthy erythrocytes (hRBCs), the active flickering deformations fall within the standard deviation measured from the population PDF ($\Sigma_{hRBC}=25\pm 4\;\text{nm}$ for $\phi p_{on}\gg 0$), aligning with the effective elasticity of living RBCs (${\bar{G}}_{hRBC}\equiv \frac{k_{B}T}{\Sigma_{hRBC}^{2}}=6.4\pm 1.2\mu N/m$). The largest flickering displacements were linked to kicking events at the hottest softened spots, estimated to be more than twice the typical deformations (i.e., for $p_{on}=1$, $\left|{\delta h}_{hRBC}^{\left(hot\right)}\right|\geq \left|\Lambda_{0}\right|\equiv 2\Sigma_{hRBC}\approx 50\;\text{nm}\gg L$; see *central panels* in Fig. 2 *B*, *left*). In rigidized erythrocytes treated with a near-toxic dose of β-escin (4 *μ*M), we observed much smaller deformation amplitudes, indicated by a narrower PDF compared with untreated cells (i.e., for $p_{on}\ll 1$, then $\Sigma_{escin}\approx 14.5\;\text{nm}\ll \Sigma_{hRBC}$; Fig. 2 *B*, *right*), demonstrating strong membrane rigidity compared with the softness of untreated cells completely active (${\bar{G}}_{escin}\equiv \frac{k_{B}T}{\Sigma_{escin}^{2}}\approx 20\;\mu N/m\gg {\bar{G}}_{hRBC}$). This escin-induced flicker hindering is similar to the passivation seen in living RBCs under activity inhibition, either metabolic by drugs (39,40), or mechanical by optical tweezers (45). The narrowest flickering distribution was seen in the case of complete membrane rigidization after extensive chemical protein cross-linking ($\Sigma_{0}\approx 8\;\text{nm}$; see Fig. 2 *A*, *right*), corresponding to the basal passive rigidity of the erythroid membrane; this is $G_{0}\equiv \frac{k_{B}T}{\Sigma_{0}^{2}}\approx 62\;\mu N/m$ (40).

#### Mobility-deformability maps: Kinetic versus potential energy

**Figure 3:**
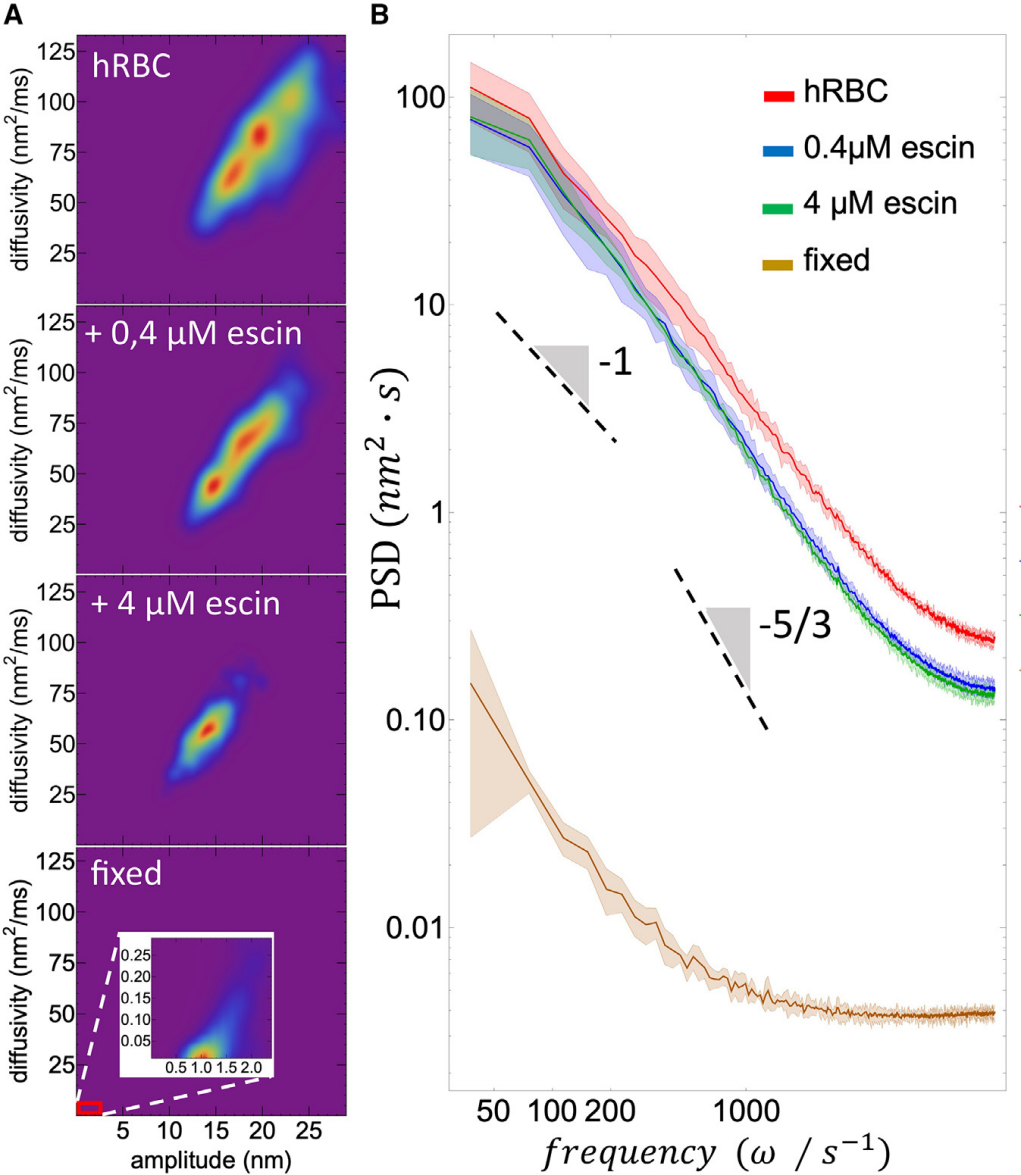
Flickering kinetics in living red blood cells treated with β-escin. (*A*) Amplitude-diffusivity density maps for discocyte populations (M≥15 individuals); appearing from top down: group 1) healthy untreated cells (hRBC) with a floppy membrane leading active kicking of high mobility under active softness (*yellow sector* for $D_{kick}$); group 2) cells treated with 0.4 *μ*M of β-escin giving rise to moderately rigidized membranes for a low dose adaptable under cell viability; group 3) cells treated with 4 *μ*M of β-escin leading a rigidized membrane for a high dose at the onset of cell toxicity; group 4) dead cells (dRBC) with a maximal membrane rigidity achieved under protein cross-linking. Such phase space mapping represents the concentration and distribution of flickering states thermalized at an effective mean temperature ${\bar{T}}_{eff}\left(\phi \right)$ for each single RBC ensemble (considering an active fraction of nonequilibrium flickering states $\phi =N_{m}/N$ detected over the N=2048 emplacements for every cell contour in M cells). The temperature scale represents the density of states in each cell population (*reddish colors* indicate hotter more populated states, and *blueish colors* colder less-populated states appearing with a lower probability). By following decreasingly lower deformation amplitudes under membrane compacting β-escin, a correlated decrease of diffusivity evidences the progressive decline of kinetic energy as decreasing ${\bar{T}}_{eff}\left(\phi \right)$ (see Eq. 2; *horizontal arrows*; group 1: high ϕ(↑); group 2: decreasing ϕ(↘); group 3: passivated ϕ(↓); group 4: passive ϕ=0). (*B*) PSDs (as population averaged values and variability band) expressed in terms of flickering frequency in living RBCs. The spectra evidence a decrease in both active direct forces (slope −1) and Brownian forces (slope −5/3), from the highest power in the healthy status (hRBC: high ${\bar{T}}_{eff}$ at ϕ(↑)) under progressive escin treatment (making ${\bar{T}}_{eff}$ decreasing at ϕ(↘)), toward membrane passivation as thermal flickering referred to the rigidized “dead” cell status at equilibrium (${\bar{T}}_{eff}=T$ at ϕ=0). Correlated active components appear at low kicking frequencies ($\omega \approx \omega_{A}\approx 5\;\text{Hz}$) decaying into a pink noise spectrum (${PSD}^{\left(act\right)}\;∼\;p_{on}f_{0}\Lambda_{0}/\omega$ for $\omega_{A}≲\omega <\omega_{D}\approx 20-50\;\text{Hz}$). Above a viscoelastic crossover depending on ${\bar{T}}_{eff}$, an intermediate diffusive regime is undergone as Brownian motions correspond to membrane softness (${PSD}^{\left(Brown\right)}∼k_{B}{\bar{T}}_{eff}/\omega^{5/3}$ for $\omega >\omega_{D}$). The spectral baseline appears as white noise that delimits experimental uncertainty at an instrumental cutoff (${PSD}^{\left(0\right)}\;∼\;\omega^{0}$ at $\omega \approx \omega_{c}\gg 500\;\text{Hz}$). For the dead cells, the passive Brownian (thermal) spectra drop under basal rigidness (i.e., ${\bar{T}}_{eff}=T$ and ${\bar{G}}_{eff}\left(T\right)=G_{0}$). See main text for details.

Fig. 3*A* shows population mappings of flickering mobility determined by local diffusion coefficients plotted against corresponding deformation amplitudes, $D_{i}\left(l_{i}\right)-\sigma_{i}\left(l_{i}\right)$, measured across many specimens procured from multiple samples (M≫20). Membrane diffusivities were calculated in four experimental groups to test the adaptability of the RBC membrane, compared with the extreme case of dead cells (see caption for details). The diffusion heatmaps revealed heterogeneous dynamics characteristic of active flickering, hence showing nonequilibrium diffusion under kicking activity in untreated living cells (group 1). Two population bumps appear on the RBC heatmaps, consistent with the expected bimodal distribution of membrane flexural modes, which include intrinsic surface tension and bending contributions to effective elasticity $G_{i}^{\left(eff\right)}$ (see Eqs. 6 and 7). Active flickering progressively decreased in escin-treated RBCs (groups 2–3) and transitioned to completely passive fluctuations in the most rigid membranes of dead cells (group 4). The gradual suppression of high-diffusivity states with increasing rigidity suggests an active origin for the highest modes, where cytoskeletal kicking is strongly hindered by membrane stiffness. These large deformation contributions are completely absent in passive RBCs with a cross-linked cytoskeleton unable to biologically adapt mechanical changes (group 4). In the velocity-deformation heatmaps, the most populated microstates corresponded to favorable mobility leading to effective kinetic energy ($K_{i}\left(v_{i}^{2}\right)\approx k_{B}T_{eff}^{\left(i\right)}/2$), linked to actively optimal elasticity described by the effective potential energy $\left(U_{i}\left(\delta h_{i}\right)\approx G_{i}^{\left(eff\right)}\sigma_{i}^{2}/2+o\left(\sigma_{i}^{4}\right)\right)$. These states involve passive (thermal) and active (propulsive) components operating under a higher effective temperature, i.e., $T_{eff}^{\left(i\right)}=T+p_{on}f_{0}\Lambda_{0}/k_{B}>T$ (with $f_{0}\Lambda_{0}>0$) (35,43); here, $p_{on}\geq 0$ represents the probability of the kicker becoming activated as hot spots, and $\pm f_{0}$ the direct kicking forces causing respective elongations $\pm \Lambda_{0}$. When fully inactivated ($p_{on}=0$), no hot spots are detected anymore in the passivated RBCs (see also Fig. 2 *B*, *right panels*).

Energy conservation can establish a reliable connection between flickering mobility and deformability under effective temperature, causing active softness. To interpret the experimental flickering mobility in relation to membrane deformability, we exploit upon the Stokes-Einstein relationship:

(12) $$D_{i}\left(l_{i}\right)\equiv \frac{k_{B}T_{eff}^{\left(i\right)}}{\zeta_{i}}\Leftrightarrow \frac{k_{B}T}{\zeta_{i}}+p_{on}\frac{f_{0}\Lambda_{0}}{\zeta_{i}}$$

This refers to the local effective temperature as a combination of passive diffusivity and active propulsion by kicking impulses, both under local friction. The local friction coefficient ($\zeta_{i}\approx 6\pi L\eta_{i}$) applies to each membrane element of size L diffusing in a local medium with effective viscosity $\eta_{i}\text{.}$ Therefore, we predict an ensemble-averaged relationship for mean flickering mobility $\bar{D}\equiv N^{-1}\sum_{i}^{N}D_{i}\propto {\bar{D}}_{eff}+\phi D_{kick}$, where the effective thermal diffusivity ${\bar{D}}_{eff}\equiv N^{-1}\sum_{i}^{N}G_{eff}^{\left(i\right)}\sigma_{i}^{2}/\zeta_{eff}^{\left(i\right)}\Rightarrow {\bar{G}}_{eff}\Sigma^{2}/{\bar{\zeta }}_{0}$ is modulated by the kicking activity $D_{kick}\approx f_{0}\Lambda_{0}/\zeta_{0}$ (given the activated fraction $\phi =N_{m}/N$) (see data in Fig. 3 *A*, *top*).

#### Dissipated power: Flickering modes

We conducted spectral analysis to examine the distribution of flickering energy across membrane deformation modes (see stochastic mechanics). Based on previous studies of active RBC flickering (39,40), we theoretically established an active bimodal fluctuation spectrum, assuming effective viscoelasticity that includes tension and bending modes affected by kicking activity (as derived from Eq. 9) (39,70):

(13a) $$PSD\left(\omega ;\phi \right)\equiv \frac{k_{B}T}{\omega G_{eff}\left(\omega ;\phi \right)}\approx \frac{k_{B}T}{\omega {\bar{G}}_{0}\left(\omega \right)}\left[1+\frac{\bar{\Delta G}\left(\omega ;\phi \right)}{{\bar{G}}_{0}}\right]$$

under frequency-renormalized flexural stiffness assumed dependent on kicking activity $\phi \left(p_{on}\right)$ as:

(13b) $${\bar{G}}_{0}\left(\omega ;\phi ,p_{on}\right)=\frac{4\gamma \left(\phi \right)}{\bar{R}}+12\pi {\left[2\eta^{2}\kappa \left(p_{on}\right)\right]}^{1/3}\omega^{2/3}$$

where R is the average radius, $\gamma \left(\phi \right)\leq \gamma_{0}\left(\phi =0\right)$, the activity-dependent lateral tension (introduced as an active Laplace pressure contribution to effective rigidness), $\kappa \left(p_{on}\right)\leq \kappa_{0}\left(p_{on}=0\right)$, the local bending rigidity (dependent on curvature-activation probability (35)), and η the apparent microviscosity.

The two mechanical effects—active kicking decreasing tension and softened bending elasticity—are difficult to disentangle and will be thoroughly discussed in the analysis of the experimental data. At low frequencies, below the diffusion crossover ($\omega <\omega_{D}\approx \gamma^{3/2}/\eta \kappa^{1/2}$), surface tension is expected to dominate, leading to a linear spectral decay, $PSD\left(\omega <\omega_{D};\phi \right)∼k_{B}T/\omega \gamma \left(\phi \right)$, appearing as a pink noise with amplitude proportional to the fraction of activated kickers (35). At high frequency ($\omega >\omega_{D}$), bending correlations dominate (70), with local curvature becoming the primary contribution (35), which is expected to be affected by kicking activation (43). Hence, an average flickering spectrum was expected to display bimodal activity features (Fig. 3 *A*), not only mechanical (demonstrated by the presence of enhanced tension and bending modes), but also metabolic as including the mean effective softness determined by the active kicking fraction (i.e., ${\tilde{G}}_{eff}\left(\omega ;\phi \right)={\bar{G}}_{0}\left(\omega \right)-\bar{\Delta G}\left(\omega ;\phi \right)$ with $\bar{\Delta G}\left(\omega ;\phi \right)/{\bar{G}}_{0}\approx \phi f_{0}\Lambda_{0}/k_{B}T\geq 0$) (35,43). In terms of the mean effective temperature, ${\bar{T}}_{eff}\left(\phi \right)=T\left(1+\phi \bar{\Delta G}/{\bar{G}}_{0}\right)$, and referred to the bare rigidness (${\bar{G}}_{0}$; Eq. 12), the flickering power spectrum can be rewritten as:

(14) $$PSD\left(\omega ;\phi \right)\equiv \frac{k_{B}{\bar{T}}_{eff}\left(\omega ;\phi \right)}{\omega {\bar{G}}_{0}\left(\omega \right)}\approx \frac{k_{B}T}{\omega {\bar{G}}_{0}\left(\omega \right)}\left[1+\phi \left(p_{on}\right)\frac{{f_{0}\Lambda }_{0}}{k_{B}T}\right]$$

where the perturbative term represents the active component proportional to the cytoskeletal kicking force (35), appearing as a low-frequency (pink noise) global enhancement to the experimental flickering spectrum (72). The leading term reflects the apparently enhanced Brownian diffusivity of the flexible membrane as dominated by effective softness across intermediate frequencies (39,40).

Fig. 3*B* illustrates that untreated living cells show overall membrane softness. When ϕ≫0, the effective temperature ${\bar{T}}_{eff}=T+\phi f_{0}\Lambda_{0}/k_{B}\gg T$, increases significantly, indicating active softness due to kicking impulses. These active deformations are mostly observed at low kicking frequencies corresponding to metabolic ATP turnover (at $\omega ≲\omega_{A}\equiv D_{kick}/\Lambda_{0}^{2}\approx 50\;s^{-1}$; *upper spectra*) (39,40). Conversely, fully rigidized (dead) cells display thermal fluctuations considered passive across all frequency ranges. For both untreated and escin-treated living cells, kicking deformations are observed in the low-frequency spectral domain (for $\omega_{A}<\omega <\omega_{D}\equiv \bar{D}/\Sigma^{2}\approx \bar{G}/\bar{\eta }\approx 500\;s^{-1}$). Here, the pink noise spectrum reflects correlated fluctuations governed by the driving force of the propulsive kickers, i.e., ${PSD}_{kick}\approx D_{kick}/\omega^{2}∼\phi \left(p_{on}\right)f_{0}\Lambda_{0}/\omega$ (39,40,42,45). In previous works, we demonstrated that normal Brownian diffusion cannot accurately describe active RBC diffusivity (40,45). Instead, living RBCs exhibit a sequence of anomalous active diffusivities within an effectively softened membrane (38,41). We identify an intermediate rheological regime of anomalous diffusivity that dominates at high frequencies beyond the viscoelastic crossover (for $\omega >\omega_{D}\approx \bar{G}/\bar{\eta }$), up to the high-frequency cutoff dominated by viscous friction (at $\omega_{C}\approx 2000\;s^{-1}$). In this range, flickering occurs under anomalous (active) Brownian motion in a flexible (softened) membrane (${\bar{G}}_{eff}\left(\phi \right)\approx {\bar{G}}_{0}-\phi \Delta \bar{G}\approx {\bar{G}}_{0}-\phi f_{0}\Lambda_{0}/h^{3}\leq {\bar{G}}_{0}$). Hence, active diffusivity depends on the equivalent effective temperature as stated in Eq. 12.

Under β-escin treatment of living cells, we observed a cooling effect that affects flickering modes in two ways (see Eq. 11a and b): first, at lower kicking frequencies ($\omega <\omega_{A}$), there is a significant decrease in global flickering activity as $\phi \left(p_{on}\right)$ decreases (also demonstrated as an $\omega_{D}$ shifting). Second, at intermediate diffusive frequencies ($\omega_{A}<\omega <\omega_{D}$), fluctuations decrease due to apparent membrane stiffening as $\Delta \bar{G}$ effectively decreases with lowering ${\bar{T}}_{eff}\to T$. This global membrane cooling reflects a shutdown of flicker propulsion, similar to escin-induced deactivation of kickers, as seen in spatial deformation maps. In completely rigid RBCs (ϕ=0; see Fig. 3 *A*, *bottom panel*), the passive diffusive response is pure thermal fluctuations, consistent with previous findings on RBC flickering passivation (see also Fig. 2 *B*), in agreement with previous results on RBC flickering passivation (35,40,42).

### Unraveling the impact of β-escin as a dual mechano-structural compacter on model lipid bilayer membranes

**Figure 4:**
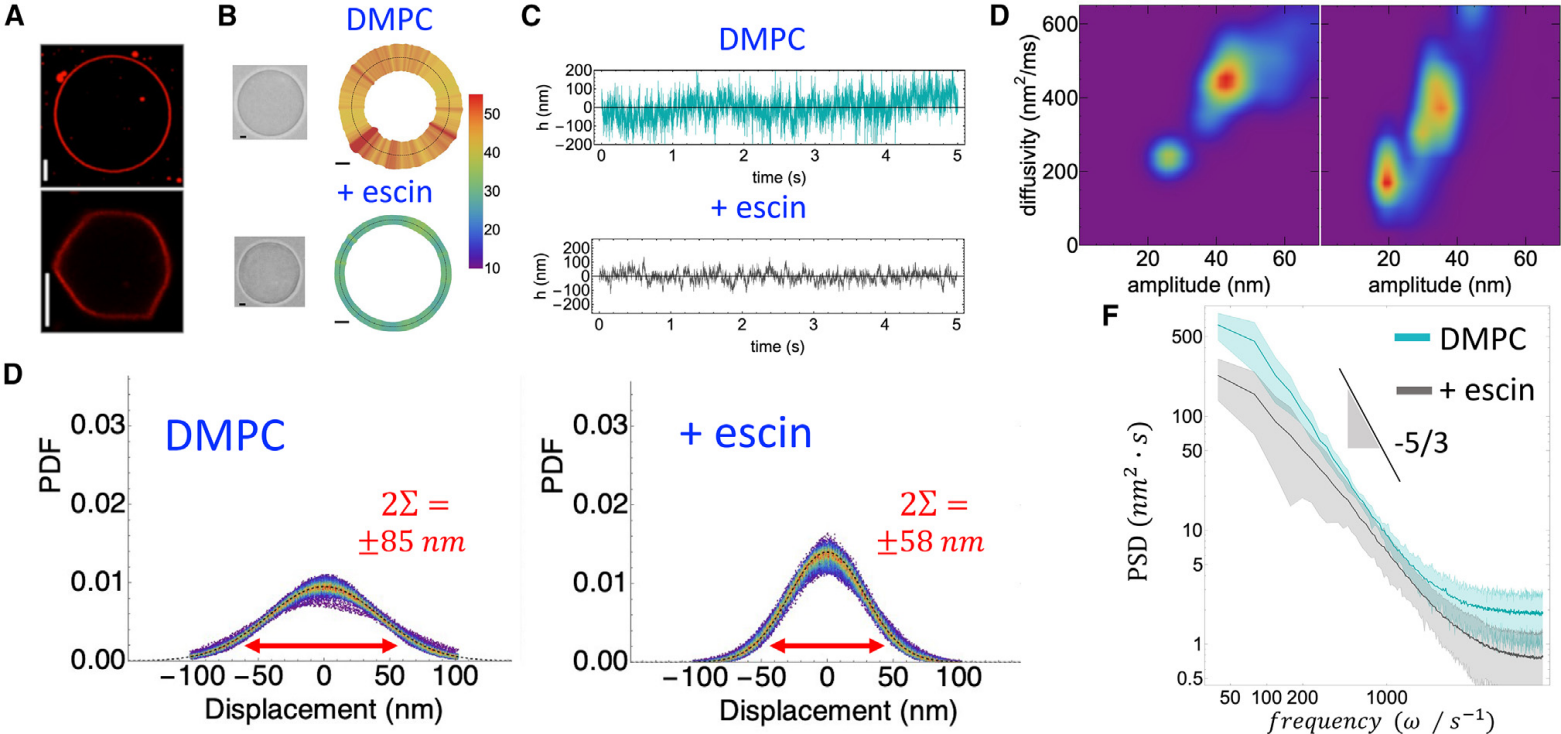
Flickering analysis in passive giant unilamellar vesicles (GUVs) made of DMPC in the presence of β-escin. (*A*) Confocal microscopy images of two representative DMPC-GUVs, tagged with RhPE, a selective fluorescent probe for disordered lipid states (pure DMPC-GUVs, *top*; DMPC-GUVs in presence of β-escin, *bottom*). Upon β-escin exposure (40 *μ*M, 1-h incubation), vesicles exhibit form alterations, indicating β-escin reorganization on the vesicle surface. (*B*) Single DMPC-GUV specimens and spatial maps of the membrane fluctuations as detected by deformation spectroscopy (scale of standard deviation). (*C*) Deformation time series as detected in equatorial emplacements. The presence of β-escin results in a reduced variance of the deformation displacements. (*D*) Probability density function (PDF) of flickering displacements for GUVs without (*left*), and with added β-escin (*right*). The presence of β-escin reduces drastically the maximum displacements (long-tailed deformations at 2Σ). (*E*) Population density distribution maps representing diffusivity versus amplitude for two GUV groups: without (*left*) and with (*right*) β-escin. The presence of β-escin decreases both membrane deformation amplitudes and fluctuation diffusivities. (*F*) PSD of GUVs without and with β-escin. The flickering power decreases as the expected Brownian spectrum of the passive (thermal) fluctuations of flexible membranes (see Eq. 4; for ϕ=0, and main text for details).

To investigate if these mechanical effects persist in passive deformations of the erythroid membrane, we studied the impact of β-escin on giant unilamellar vesicles (DMPC-GUVs), above the melting transition of the phospholipid ($T>T_{m}$) (51). Replicating the approach used for living RBCs (as shown in Figs. 2 and 3), Fig. 4 presents the same flickering analysis applied to passive GUVs. The results reveal bimodal population heatmaps similar to those observed in living RBCs but without the suppression trend, as passive GUVs cannot adapt to mechanical changes. Instead, they exhibit only broader thermal fluctuations, reflecting sample variability at the high temperature considered ($T=37ºC\gg T_{m}$).

#### Structural impact of escin on the passive flexible membranes

Fig. 4*A* shows GUVs made of pure DMPC (*top panels*) and hybrid DMPC-GUVs incubated with β-escin (*bottom panels*). The vesicles were labeled with RhPE, a fluorescent probe for phospholipids in the liquid disordered state (73). Exposed to high β-escin concentration (40 *μ*M, below the CMC of 0.35 mM (49)), the GUVs exhibited a strong structural impact, with some losing their spherical shape and becoming polyhedral. This change is due to β-escin’s compacting effect on the outer membrane surface (48,51,52,53). Fig. 4 *B* shows spatial maps of equilibrium fluctuations in GUVs, composed of fluid bilayers without a structural skeleton to transfer nonequilibrium flickering activity (39,42). Compared with active RBC flickering, passive GUVs had higher fluctuation amplitudes but lower mechanical heterogeneity (see also Fig, 3). The fluctuation time series in Fig, 4 *C* show that passive membrane deformations are visibly hindered by β-escin, indicating its stiffening effect on the model membranes (48,51,52,53). Fig. 4 *D* presents the ensemble-averaged PDFs for both scenarios, revealing maximum membrane deformations of about 85 nm for the bare DMPC membrane (*left panel*), which decrease to about 58 nm in the presence of β-escin (*right panel*).

#### Diffusive membrane dynamics

In Fig. 4 *E*, we present diffusivity-deformation density maps for the two groups of DMPC-GUVs studied. The ensemble distributions showed more diffusion in the more flexible GUVs compared with RBCs, which have a structural spectrin skeleton (Fig. 3 *C*). The insertion of β-escin in the DMPC-GUV bilayer caused membrane rigidization and a slowdown in diffusion (*left panel*). In contrast, faster diffusion and greater deformability were observed in the absence of β-escin (*right panel*). As proof of the mechanical effects of β-escin on the lipid bilayer, Fig. 4 *F* compares Brownian PSDs. These PSDs are affected by the basal elasticity of the passive membrane and by viscous friction, represented by ${PSD}^{\left(pass\right)}\approx k_{B}T/{\left(\eta^{2}G_{0}\omega^{5}\right)}^{1/3}$ (39). When β-escin was incorporated into the DMPC-GUVs, there was a significant decrease in passive flickering power, indicating a hindrance of thermal fluctuations due to membrane compaction (increasing $G_{0}$ and probably η).

### Rheological analysis: Effective softness and viscosity

To better understand the mechanical interactions between β-escin and lipid bilayers and their effects on membrane fluctuations, we conducted a rheological analysis across all studied cases (RBCs and DMPC-GUVs). Our approach is based on passive particle tracking microrheology (74), using mean square displacements expressed in frequency space (Fourier) through the calculated PSDs (see Eq. 11a and b). The viscoelastic memory function can be expressed as a generalized complex impedance (${\tilde{G}}_{eff}^{\left(i\right)}=G+i\omega \eta_{i}\Rightarrow \omega {\tilde{\eta }}_{i}$), which is locally connected to the local diffusivity coefficient ${\tilde{D}}_{i}=k_{B}T/6\pi L{\tilde{\eta }}_{i}$ (75,76), thus relating to the flickering mobility through the autocorrelation function of the local velocities (see stochastic mechanics). From the generalized Stokes-Einstein relationship, an effective rheological impedance holds under environmental temperature (i.e., by taking ${\bar{T}}_{eff}=T$ in Eq. 10):

(15) $${\tilde{G}}_{eff}\left(\omega \right)=\frac{1}{6\pi L\omega }\frac{k_{B}T}{\left\langle \delta h^{2}\left(\omega \right)\right\rangle }$$

as calculated from the experimental deformations in frequency space $\left\langle \delta h^{2}\left(\omega \right)\right\rangle =2\tilde{D}/\omega^{2}$ (68,74).

The rheological memory function was given as ${\tilde{G}}_{eff}\left(\omega \right)\equiv G_{eff}+i\omega \eta_{eff}$, indicating each membrane location at a given flickering frequency (ω) (45). Here, the real part represents the mean active softness referenced to the passive flexibility of the membrane minus the active softening term (${\bar{G}}_{eff}={\bar{G}}_{0}-\Delta \bar{G}\leq {\bar{G}}_{0}$), and the imaginary part the effective viscosity with respect to the bulk fluid (${\bar{\eta }}_{eff}={\bar{\eta }}_{0}+\Delta \bar{\eta }\geq {\bar{\eta }}_{0}$). Hence, for optimal softness regulation with minimal dissipation, we expect $\Delta \bar{G}>0$ and $\Delta \bar{\eta }\approx 0$. Our goal was to determine the mean rheological changes caused by the compacting integration of β-escin into the lipid membrane, specifically the effective values of flexural rigidity (${\bar{G}}_{eff}$) and microviscosity (${\bar{\eta }}_{eff}$). These rheological parameters are crucial for understanding membrane flickering dynamics and flexibility, which are essential for cellular functionality, especially under adaptive conditions.

#### Rheological mappings: Active flickering heterogeneity

**Figure 5:**
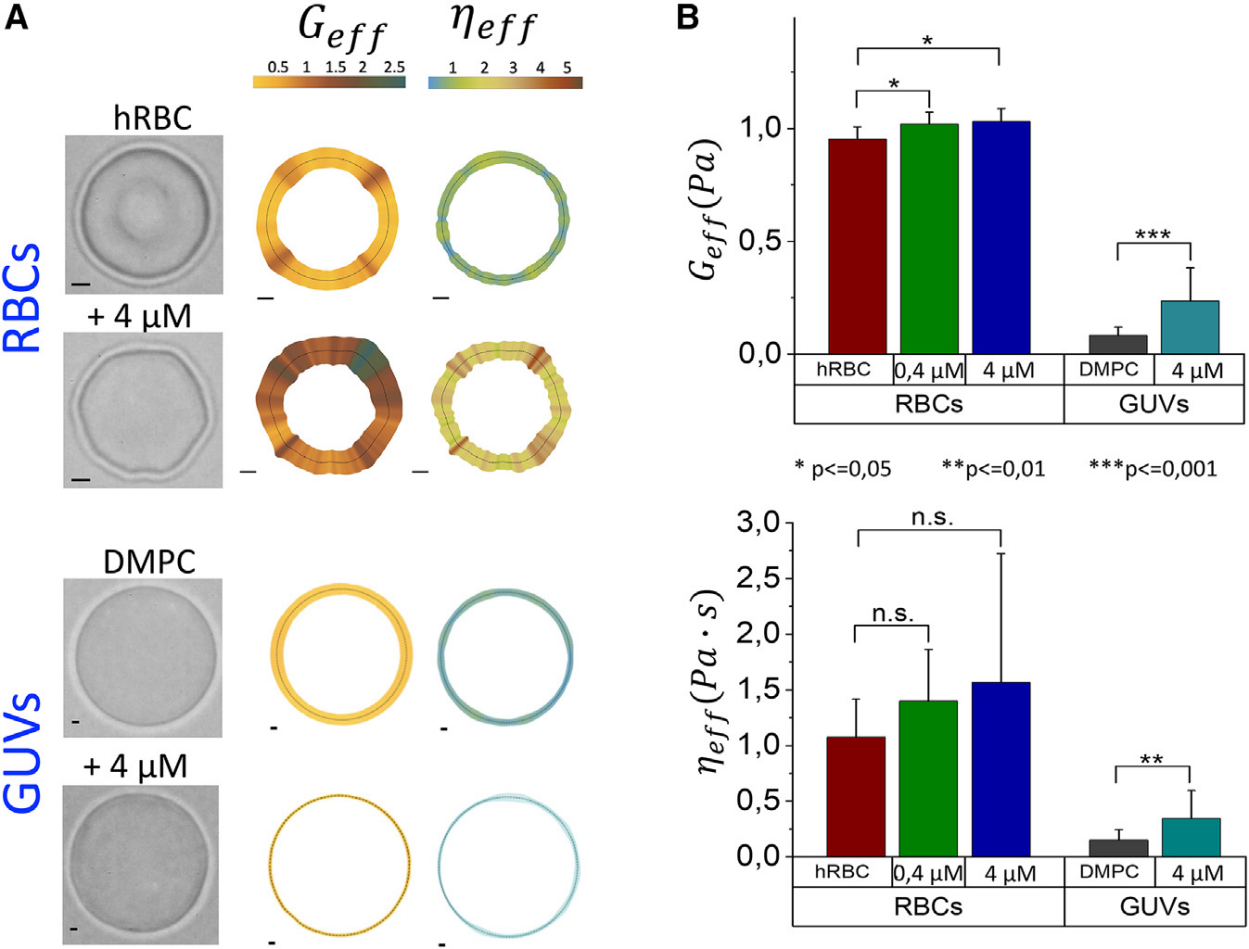
Compared rheological properties of erythrocytes and DMPC vesicles in the absence and presence of β-escin. (*A*) Spatial rheological maps as local distribution profiles of flexural stiffness (${\bar{G}}_{eff}$) and microviscosity (${\bar{\eta }}_{eff}$) for RBCs in the presence of β-escin (RBC + 4 *μ*M), healthy cells (hRBC), DMPC vesicles treated with 40 *μ*M β-escin (DMPC + 40 *μ*M), and pure DMPC vesicles (pure DMPC). (*B*) Population statistics for the effective rheological properties. The boxplots represent the calculated stiffness (*upper plot*) and viscosity (*lower plot*) for each group (M>20 specimens): DMPC vesicles (L), DMPC vesicles in the presence of 40 *μ*M β-escin (L + 40 *μ*M), healthy red blood cells (hRBC), RBCs treated with 0.4 *μ*M β-escin (+0.4 *μ*M), and RBCs treated with 4 *μ*M β-escin (+4 *μ*M).

Fig. 5 shows representative spatial mappings of living RBCs and GUV models exposed to a high dose of β-escin (Fig. 5 *A*; also discussed in Figs. 1, 2, 3, and 4), allowing for a visual comparison with statistically significant population datasets (Fig. 5 *B*). Notably, regulated membrane softness and viscosity control were observed in living RBCs under β-escin treatment. The active RBC membrane shows higher elastic heterogeneity than the passive unregulated GUVs, which undergo a significant increase in flexural rigidity under escin exposure (Fig. 5 *B*). The altered rigidity profiles indicated increased mechanical heterogeneity in RBCs, while their membrane microviscosity increased only slightly and more homogeneously compared with changes in the flexural modulus. This mechanical heterogeneity was more pronounced when compared with untreated RBCs, which—although dynamically heterogeneous—displayed less variability than the treated cells (Fig. 5 *B*). In contrast, the DMPC-GUVs—representing passive lipid membranes with a nonadaptive mechanics—showed no discernible heterogeneity in either the spatial mechanical maps (Fig. 5 *A*) or the statistical population distributions (Fig. 5 *B*). The observed values of membrane rigidity and viscosity for DMPC-GUVs were found greatly smaller than those for RBCs, consistent with expectations for lipid-only constructs devoid of a functional cytoskeleton. Indeed, the crucial mechanical difference between living adaptive RBCs and dead nonadaptive GUVs lies in their observed heterogeneous membrane responses to structural perturbations induced by β-Aescin (51). Living RBCs exhibit regulated heterogeneity (45), maintaining near-constant average viscoelastic properties here revealed under escin treatment. This adaptive behavior allows living RBCs to control membrane effective softness and viscosity (40,45), preserving functional flexibility despite mechanical stress induced by escin. In contrast, nonadaptive GUVs, representing passive lipid membranes, lack this regulatory capacity (51). Here, we reveal them to exhibit a uniform mechanical response under escin treatment, with increased rigidity and no spatial heterogeneity, highlighting their purely passive nature. The presence of a functional cytoskeleton in living RBCs is essential for their dynamic adaptability, an activity feature absent in GUVs, which behave as passive lipid constructs incapable of modulating their mechanical state across dynamical scales. A deeper analysis of dynamic heterogeneity follows on the observed viscoelastic memory functions.

#### Active RBC membrane rheology: Regulated softness and fluidity

**Figure 6:**
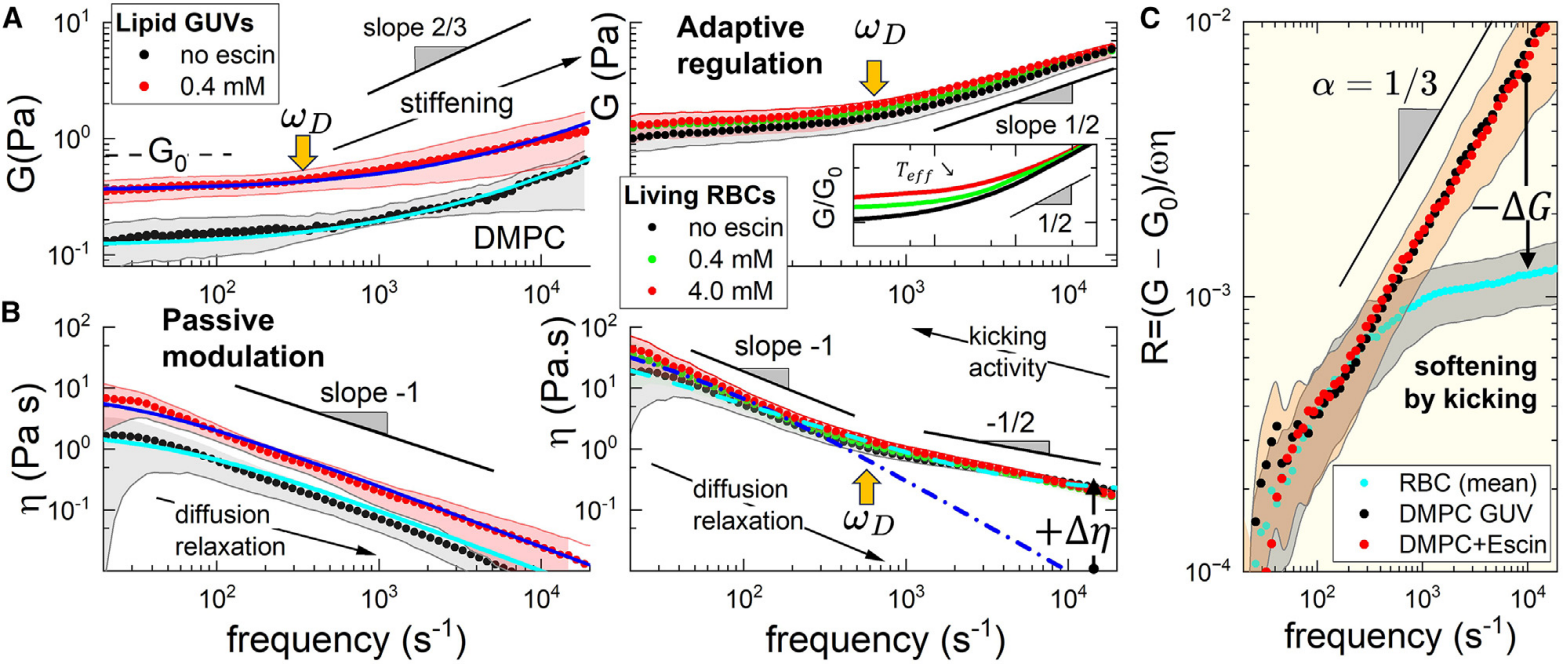
β-escin impact on rheological memory functions compared with an anomalous diffusion model (described in Eqs. 14, 15, and 16, under fractional scaling exponent, 0≤α≤1). (*A*) Flexural modulus ($G_{eff}∼\omega^{2\alpha }$ in *top panels*; l*eftmost* for DMPC GUVs; *right* for living RBCs). (*B*) Shear microviscosity ($\eta_{eff}∼\omega^{-2\alpha }$ in *bottom panels*; same cases as in *A*), both plotted as population averages across all studied groups (*legends* indicate escin concentration in the incubation medium). Passive lipid GUVs (*left panels*); under escin incorporation (*symbols:* dots are experimental data; s*traight lines* represent best fittings to passive fractional model, ϕ=0; Eq. 14 for $G_{eff}$; Eq. 16 for $\eta_{eff}$). DMPC-based GUVs exhibit passive rheology modulated by β-escin, from a quasistatic response ($G_{0}$ and $\eta_{0}$) up to fractional (heterogeneous) above the diffusive frequency $\omega_{D}\equiv \tau_{D}^{-1}$ (*left panels*; at $\omega >\omega_{D}$, experimental data display dependence on escin-concentration with dynamic memory functions $G_{eff}∼\omega^{2/3}$ ]*top*], and $\eta_{eff}∼\omega^{-1}$ [*bottom*], hence α≈1/3, for ϕ=0). Active living RBCs (*central panels*); these metabolically active viscoelastic biomembranes exhibit anomalous diffusion under active flickering upon effective temperature ($T_{eff}>T$ for ϕ>0), leading to regulated fractional rheology characterized by a dynamic response not dependent on the presence of β-escin. Living RBCs exhibit adaptive behavior through active viscoelastic regulation (ϕ>0), maintaining a controlled rigidity modulus and microviscosity across diffusive scales above the characteristic active (kicking) rate ($\omega_{A}<\omega \approx \omega_{D}$). (*Top*) Adaptive elasticity as predicted for fractional Maxwell relaxation under active anomalous diffusivity (ϕ>0 and α≈1/4). (*Inset*) Straight lines represent best fittings of experimental data to Eq. 14 (variable $T_{eff}\left(\phi \right)$; *bottom-up*): no escin, active fractional Maxwell-fluid (ϕ=0.35±0.12, α=0.22±0.15; *black line*); 0.4 mM added escin, fractionally passivated fluid (ϕ=0.21±0.15, α=0.22±0.10; *green line*); 4 mM escin, near passive solid (ϕ=0.08±0.12, α=0.27±0.14; *red line*). (*Bottom*) Adaptive behavior reflects a linear viscosity decrease in the lubrication limit at subdiffusive frequencies ($\eta_{pass}∼\omega^{-1}$ at $\omega <\omega_{D}$), renormalizing to an actively dissipative regime in the high-frequency kicking domain ($\eta_{act}∼\omega^{-1/2}$ at $\omega >\omega_{D}$), resulting in effective viscoelastic regulation (see main text for details). (*C*) Viscoelastic ratio under fractional memory function (mean values for different systems; β-Aescin treatment). Passive GUVs exhibit confined Brownian diffusivity (α=1/3), governed by structural correlations at high frequencies. Living RBCs display anomalous diffusivity (even after β-Aescin treatment), regulated by active kicking forces (α→0). Active softening (−ΔG) appears evident as a drop in the viscoelastic ratio between passive fluctuating systems (ϕ=0) and active flickering systems (ϕ>0).

Fig. 6 presents a detailed rheological analysis of passive DMPC-GUVs (*left panels*) and active RBCs (*central panels*). The experimental values for the effective rigidity modulus, $G_{eff}\left(\omega \right)$ (*upper panel*), and effective viscosity, $\eta_{eff}\left(\omega \right)$ (*lower panel*), are shown as functions of frequency (ω), calculated using the Mason and Weitz microrheological method (74) (see (13a), (13b)). The observed rheological regimes align with an effective flexural modulus, ${\bar{G}}_{0}\left({\bar{T}}_{eff}\right)={\bar{G}}_{\gamma }+{\bar{G}}_{\kappa }=4\gamma_{eff}/R+12\pi {\left(d/R\right)\left(2\eta^{2}\kappa_{eff}\right)}^{1/3}\omega^{2/3}$ (see Eq. 14), consistent with the expected renormalization (39,70) under anomalous diffusivity (77). Fig. 6 *A* illustrates the diffusive rheological crossover (at $\omega_{D}\approx 300-400\;s^{-1}$), which is barely detectable in passive DMPC-GUVs (*left panel*). This dynamic transition marks a rigidity shift from the surface tension regime (frequency independent, ${\bar{G}}_{pass}^{\left(\gamma \right)}∼\gamma \omega^{0}$), up to the bending regime (frequency dependent $,{\bar{G}}_{pass}^{\left(\kappa \right)}∼\kappa_{0}^{1/3}\omega^{2/3}$), which increases (unregulated) with adding β-escin ($\kappa_{0}$ reases with escin concentration (51)). In living RBCs, this mechanical signature persists, appearing, however, as a regulated renormalization from static rigidity to an anomalous subdiffusive bending regime at high frequencies (${\bar{G}}_{act}^{\left(\kappa \right)}∼\omega^{1/2}$ with constant κ, independent of escin concentration). This anomalous subdiffusivity was previously identified in displacement variances measured below the confinement plateau (45). Fig. 6 *B* presents microviscosity data, showing a linear rheological decay ($\eta ∼\omega^{-1}$), characteristic of a lubrication regime (constant $\omega \eta_{pass}$), indicative of frictional losses in passive GUVs. In contrast, living RBCs exhibit a renormalized response, transitioning from low-frequency passive lubrication below the diffusive frequency ($\eta_{pass}∼\omega^{-1}$ at $\omega <\omega_{D}$), up to an active fractional regime at higher frequencies ($\eta_{act}∼\omega^{-1/2}\;\text{at}\;\omega >\omega_{D}$). Overall, passive GUVs show merely mechanical modulation upon β-escin addition, marked by significant membrane stiffening and increased viscosity—both consistent with previous rheological data demonstrating dual (fluid- or solid-like) membrane viscoelasticity depending on shear deformation (51) (Fig. 6, *left panels*). In contrast, active RBCs display adaptive regulation, maintaining a mechanical structure akin to passive membranes but effectively modified by coupled diffusive mechanisms under an effective temperature (Fig. 6, *central panels*). These rheological results suggest an apparent Maxwell relaxation encompassing a viscoelastic crossover at the diffusive frequency ($\omega_{D}\equiv D_{eff}/\Sigma^{2}\Rightarrow G_{eff}/\eta_{eff}$).

On the one hand, the mean effective rigidity increased as deformation frequencies entered the diffusive domain, indicating membrane softness controlled by viscoelastic relaxation (at $\omega >\omega_{D}\approx 200-500\;s^{-1}\gg \omega_{A}$). Furthermore, the viscoelastic relaxation is considered diffusively fractional (77), effectively renormalizing the curvature diffusive motions driven by global kicking forces (35,43). Consequently, the effective rigidity modulus can be formulated as:

(16) $${\bar{G}}_{eff}\left(\omega ;\phi \right)\approx \frac{\gamma \left(\phi \right)}{R\left(h\right)}+{\bar{G}}_{\kappa }\left[\phi \left(p_{on}\right)\right]\frac{{\left(\omega \tau_{D}\right)}^{2\alpha }}{1+{\left(\omega \tau_{D}\right)}^{2\alpha }}$$

Unlike passive Brownian diffusivity (α=1), active membrane deformations under such effective rigidity follow an anomalous diffusion law—the mean square flickering displacement, ${MSD}_{anom}=2D_{\alpha }\tau^{\alpha }$ (with α<1), where $D_{\alpha }\left(\omega \right)=D\tau_{D}/{\left(\omega \tau_{D}\right)}^{\alpha }$ is the generalized diffusion coefficient fixed by the diffusive time $\tau_{D}\equiv \omega_{D}^{-1}$ (77). At low frequencies ($\omega \tau_{D}<1$), the tension term always dominates, ${\bar{G}}_{\gamma }\left(\phi ;\omega \to 0\right)\propto \omega^{0}$. At high frequency ($\omega \tau_{D}>1$), the bending component emerges for passive subdiffusivity (${\bar{G}}_{pass}^{\left(\kappa \right)}\left(\phi =0;\omega >\omega_{D}\right)\propto \omega^{2/3}$), as observed for passive GUVs, taking α=1/3 (70) (Fig. 6 *A*, *left panel*).

Within this heuristic model, global flickering modes are actively controlled by the apparent membrane tension γ(ϕ>0), which can be overcome by the relaxation strength from local (mesoscopic) diffusive modes under ${\bar{G}}_{\kappa }={\bar{G}}_{pass}^{\left(\kappa \right)}-\bar{\Delta G}\left[\phi \left(p_{on}\right)\right]$. The effective flexural rigidity emerges from a phenomenological balance between tension-driven (Laplace) pressure, which defines the static rigidity as ${\bar{G}}_{\gamma }\left(\phi ;\omega \to 0\right)=\gamma \left(\phi \right)/R$, followed by an apparently diffusive (Maxwell-like) relaxation modulated by an effective bending softness ${\bar{\Delta G}}_{\kappa }\left(\phi ;\omega \right)={\bar{G}}_{\kappa }\left[{\bar{T}}_{eff}\left(\phi ;\omega \right)/T-1\right]$, under frequency-dependent effective temperature (43):

(17) $${\bar{T}}_{eff}\left(\omega /\omega_{A};\phi \right)\approx T+\phi \left(p_{on}\right)\frac{f_{0}\Lambda_{0}}{k_{B}}\frac{1-\cos\left(\omega \tau_{A}\right)}{{\left(\omega \tau_{A}\right)}^{2}}$$

which is metabolically controlled by the unitary energy of the cytoskeletal kickers ($\epsilon_{kick}=f_{0}\Lambda_{0}\gg k_{B}T$), and their characteristic pulsation time ($\tau_{A}>\tau_{D}$), under activity fraction $\phi \left(p_{on}\right)$ (43).

For RBC flickers at low frequencies ($\omega \tau_{A}<1$), if $\phi \left(p_{on}\right)\gg 0$, the effective tension term appears dominant under a constant high effective temperature, ${\bar{T}}_{eff}\left(\phi \right)\approx T+\phi \left(p_{on}\right)\frac{f_{0}\Lambda_{0}}{k_{B}}\gg T$, hence resulting into a regulated rigidity (i.e., constant ${\bar{G}}_{\gamma }\left[\;{\bar{T}}_{eff}\left(\phi \right)\right]$, at $\omega <\omega_{A}$) (43). However, at high frequencies ($\omega \tau_{A}\gg 1$ and $\omega >\omega_{D}$), the active softening term dominates ($\bar{\Delta G}\left(\omega \right)/{\bar{G}}_{pass}^{\left(\kappa \right)}\gg 1$), resulting in ${\bar{G}}_{\kappa }\left(\omega \right)∼\;\phi \left(p_{on}\right)f_{0}\omega^{1/2}$, as experimentally observed (Fig. 6 *A*, *central panel*). This active softness regulation was not observed in passive DMPC membranes, which displayed monotonic elasticity (if ϕ=0, then ${\bar{G}}_{eff}\approx \frac{\gamma_{0}}{R}+{\bar{G}}_{pass}^{\left(\kappa \right)}$).

The regulated flexural softness arises from a balance between tension-driven (Laplace) pressure and subdiffusive bending relaxation. At low diffusion frequencies ($\omega \tau_{D}\ll 1$), apparent rigidity follows: ${\bar{G}}_{eff}^{\left(\gamma \right)}\left(\phi ;\omega \to 0\right)=\gamma_{eff}\left(\phi \right)/R$. However, at higher frequencies ($\omega \tau_{D}\gg 1$), bending relaxation dominates flickering: ${\bar{G}}_{eff}^{\left(\kappa \right)}\left(p_{on}\right)\propto \kappa_{eff}^{1/4}\left(p_{on}\right)\omega^{1/2}$. Such active regulation of membrane stiffness is controlled by the RBC cytoskeleton (under ϕ and $p_{on}$) (43).

On the other hand, the mean effective viscosity decreased across all frequencies, corresponding to membrane thinning under frictional dissipation. For living RBCs, there was a near-invariant viscosity above the diffusive threshold (from $\omega >\omega_{D}\approx 1-2\;\text{kHz}$ up to higher flickering frequencies). Escin-treated RBCs showed progressively lower viscous friction within the intermediate frequency domain (at $\omega_{A}<\omega <\omega_{D}$). This regulation persisted regardless of escin concentration. Therefore, effective viscosity can also be described through conjugate fractional relaxation (77), analogous to effective stiffness (Eq. 14), as follows:

(18) $${\bar{\eta }}_{eff}\left(\omega ;\phi \right)\approx \eta_{0}+{\bar{G}}_{0}\left[\phi \left(p_{on}\right)\right]\frac{{\left(\omega \tau_{D}\right)}^{\alpha -1}}{1+{\left(\omega \tau_{D}\right)}^{2\alpha }}+\phi \left(p_{on}\right)\frac{f_{0}^{2}}{4\eta_{0}L}$$

where $\eta_{0}$ holds for the apparent viscosity of the membrane-milieu system.

At low frequencies ($\omega \tau_{D}\ll 1$), the frictional relaxation term is dominated by membrane microviscosity, $\eta_{0}\left[\phi \left(p_{on}\right)\right]$, under fractional decay dependent on diffusivity; for passive GUVs (ϕ=0 and α=1); for living RBCs (ϕ≫0 and α<1). At high frequencies ($\omega \tau_{D}\ll 1$), if ϕ≫0, a dissipative component emerges due to the active kickers’ pulsation against the solvent viscosity, given by $\Delta {\bar{\eta }}_{eff}\approx \phi \left(p_{on}\right)\frac{f_{0}^{2}}{4\eta_{0}L}$. This active thickening term counteracts structural viscous thinning. Of course, this active regulation does not occur in passive DMPC membranes, which show pure Maxwell-like viscous dissipation, i.e., ${\bar{\eta }}_{eff}\left(\omega ;\phi =0\right)\approx {\bar{\eta }}_{0}+{\bar{G}}_{0}\frac{\omega_{D}}{\omega^{2}+\omega_{D}^{2}}\ll {\bar{\eta }}_{eff}\left(\omega ;\phi \gg 0\right)\text{.}$

Therefore, we have linked the observed rheology to the effective rigidity function, ${\bar{G}}_{eff}\left(\omega ;\phi \right)={\bar{G}}_{0}\left({\bar{T}}_{eff}\right)-\bar{\Delta G}\left[\phi \left(p_{on}\right)\right]$, incorporating diffusional relaxation under fractional exponent, α, and active softness defined by the effective temperature, ${\bar{T}}_{eff}$ (Eqs. 14, 15, and 16). The experimental data have been fitted to the fractional active model, with the best-fit plots included in the different panels of Fig. 6. More specifically, a detailed analysis of the effective viscoelastic ratio in Fig. 6 *C*, $R_{eff}={\bar{G}}_{eff}/{\bar{\eta }}_{eff}$, reveals the confined softness dynamics underlying the active flickering mechanism in living RBCs, in contrast to the purely structural fractional diffusivity observed in passive GUVs.

## Discussion

### RBC rheological adaptivity

We highlight the dynamic interplay between the adaptable outer lipid bilayer and its mechanobiological response under active (homeostatic) conditions set by the membrane skeleton (1,5,7,9). Active RBC flickering shows dually regulated adaptability to stresses from the mechano-active surfactant β-escin, akin to passive deformations in model membranes driven by thermal fluctuations (51). Under hardening treatment with the mechano-active surfactant β-escin, our experiments show that RBC flickers remain biologically active within physiological viability limits (see Figs. 1 and 2), demonstrating adaptive responsiveness regulated by nonequilibrium membrane processes. While the natural saponin β-escin significantly reduces thermal fluctuations in passive GUVs due to unregulated membrane hardening (see Fig. 4), this membrane hardener does not inactivate flickering in actively regulated RBCs (see Figs. 2 and 3). Instead, β-escin induces only moderate membrane stiffening while maintaining membrane fluidity in living RBCs (see Fig. 5). This homeostatic regulation supports rheological adaptability through membrane softness, preserving biological viability.

In biophysical terms, we have demonstrated that RBCs exhibit rheological adaptability, enabling spatially localized, active flickering, as previously reported under nonequilibrium conditions across various spatiotemporal scales (40,44). Notably, our current experimental results with escin-treated RBCs have revealed an adaptive homeostatic regulation of the flickering activity (Fig. 3). Specifically, we observe the living cells undertaking active flickering upon an adaptively membrane softness over a wide range of spatiotemporal scales, unlike the rigidized cytoskeleton in dead cells (see fixed cells in Fig. 3) or the passive lipid bilayers in GUVs (see Fig. 4). Furthermore, active viscoelasticity emerges at mesoscopic scales, spanning from low kicking frequencies to intermediate frequencies below the mere thermal regime (see compared data in Figs. 3 and 4). Such active rheological regulation (homeostatic) likely reflects the interaction between the escin-integrated lipid bilayer and the protein cytoskeleton. Hence, we argue the actively regulated flickering motions dissipating energy more efficiently through a softened membrane, following a minimal friction pathway relative to the passive GUVs undergoing higher dissipation through thermal fluctuations (Fig. 4). In the different experimental groups of living RBCs (reported in Fig. 3, either untreated or treated with escin), the flexible spectrin filaments and actomyosin motors of the active cytoskeleton maintain a mechanically adapted membrane softness and regulate energy dissipation across rheologically active mesoscopic scales (9,15,24). This dynamic organization allows RBCs to actively flicker while preserving their mechanical deformability, a key property for their physiological function (1,5,7,9). After β-escin treatment, localized flickering persisted mechanically adaptive at low and intermediate mesoscopic frequencies, hence supporting the overall homeostatic response.

Structurally, unlike surfactants that permeabilize cell membranes by creating local pores, β-escin adsorbs onto the outer lipid bilayer of RBCs, altering their mesoscopic rheology. In living RBCs, escin does not affect membrane microviscosity but induces slight membrane stiffening, likely by imposing adaptable flexural constraints on the cytoskeletal network without disrupting the lateral mobility of the lipid bilayer. Experimental data show that β-escin’s regulatory effect on membrane viscoelasticity leads to an apparent cooling of treated cells, even at near-toxic doses (4 *μ*M). This physiological regulation appears to be driven by global changes in effective temperature imposed by the active cytoskeleton. Our findings suggest that escin-lipid interactions, previously observed in passive models (51,52,53), contribute to the adaptive viscoelasticity mediated by active kicking motions. This process is metabolically regulated by effective temperature within the flickering diffusive domain. Our results highlight the key role of the cytoskeleton and lipid membrane in modulating the active flickering of living RBCs in response to external stresses—contrasting sharply with the passive rheology of model lipid systems lacking a cytoskeleton.

### Flickering deformation and rheological homeostasis

The hallmark of biological cell adaptability is homeostasis—the autonomous regulatory metabolism coupled with cytoskeletal effectors that drive cellular activity. These homeostatic interactions coordinate cellular dynamics, enabling adaptation to environmental changes. A key example is the homeostatic deformability of RBCs, encoded in the cytoskeleton as active shape fluctuations driven by high-temperature flickering. This adaptability arises from hierarchical power production and regulates homeostatic synergies (42), which organize heterogeneous cellular structures despite dissipation (78,79). Active RBC flickering—a nonequilibrium process—is powered by metabolic ATP consumption from glycolytic reservoirs in the cytoplasm (30,31,32). This organized stochasticity ensures that RBC flickering operates predictably within a homeostatic functional range, far from equilibrium (39,42). The nonequilibrium dynamics underlying RBC flickering facilitate recovery from disturbing deformations, enhancing adaptability to both mechanical and chemical stress, such as the reported membrane adsorption of escin. Unregulated RBC deformability following hemoglobin release due to overdosed circulating saponins may affect flickering deformations by disrupting cytoskeletal-membrane interactions and altering intracellular viscosity. This could lead to modified membrane fluctuations resulting from weakened membrane-cytoskeleton connectivity and reduced intracellular pressure. Additionally, β-escin-induced changes in membrane tension (as observed in our experiments) may further influence these active fluctuations.

### RBC membrane flickering: An adaptive rheological mechanism

On the theoretical basis of active flickering, we propose the following biophysical interpretation of the experimental evidence as an adaptive mechanism (revealed from rheological analyses in Figs. 5 and 6): escin (or other membrane biosurfactants in circulation e.g., cholesterol) increases bilayer rigidity, akin to its effect in passive GUVs, leading to a higher membrane rigidity. Consequently, the force exerted by active kickers is reduced, as kicking in RBCs depends on the spectrin network’s ability to bend and stretch the bilayer. Since the bilayer becomes more rigid, the spectrin network deforms it less effectively, thereby diminishing the ATP-driven spectrin attachment/detachment process responsible for driving the kickers. As a result, RBCs modify metabolism for making adaptive flickering dynamics within the active membrane, modulated by softening interactions at the mesoscopic scale of self-propelled kickers. Based on the reported evidence, we propose further modeling this adaptive flickering behavior using a statistical mechanics approach.

## Conclusions

In a biophysical perspective, our experimental findings revealed two distinct mechanical effects of β-escin on membrane-compacted living RBCs, leading to adaptive flickering activity by energy interconversion: 1) the conservative strength of the flickering deformations under elastic potential energy. 2) the dissipative magnitude of the flickering mobilities under diffusive kinetic energy. We have investigated whether this biomechanical regulation arises from a homeostatic adaptation of living cells, actively modulating their membrane mechanics, or if it is simply a passive consequence of membrane compaction imposing mechanical constraints. By studying model passive membranes devoid of metabolism, we aimed to distinguish between adaptive regulation and purely mechanical constraints, as explored in previous works (80,81). Our analysis of β-escin’s dynamic interaction with fluid DMPC phospholipid vesicles sought to clarify its role as both a membrane stiffener, enhancing flexural rigidity, and a thickening agent, increasing lateral viscosity. In our recent study with model phospholipid membranes (51), we found that β-escin exerts a dual mechanical effect, resembling the interaction of membrane cholesterol with lipid bilayer leaflets. In living RBCs, functional membrane flickering and active deformability are intrinsically linked to cellular and membrane metabolism, playing a key role in mechanobiological regulation within the bloodstream. Hematological disorders such as anemia, hemolytic disease, and diabetes can significantly alter RBC shape and flexibility (82), thereby affecting capillary microcirculation (83). In conditions such as chronic venous disease (CVD), disruptions in RBC rheology contribute to impaired deformability, which in turn impacts hemodynamics (84). Current CVD treatments, including natural saponins such as escin, aim to modulate red cell membrane rigidity to mitigate inflammation (85). Based on our findings on the biomechanical effects of β-escin on erythroid dynamics, we explore whether human RBCs can actively adjust their rheological properties in response to saponin-induced modifications in membrane mechanics. This perspective offers potential insights into the therapeutic applications of saponin biosurfactants in managing hemolytic disorders and vascular diseases.

## Data and code availability

The raw data that support the findings of this study are available on request from the corresponding authors.

## Acknowledgments

L.H.M. is contracted by María Zambrano Program from Ministerio de Universidades de España for the attraction of international talent under Next Generation European Union funding (grant CT19/22). The work was supported by the 10.13039/501100004837Spanish Ministry of Science and Innovation (MICINN–Agencia Española de Investigación AEI) under grants PID2019-108391RB-100 and TED2021-132296B-C52 (to F.M.), and 10.13039/100012818Comunidad de Madrid under grants S2018/NMT-4389 and Y2018/BIO-5207 (to F.M.). We also acknowledge the financial support of the 10.13039/501100001659German Research Foundation DFG grant HE 2995/7-1 (to T.H.). This study was also funded by the REACT-EU program PR38-21- 28 ANTICIPA-CM, a grant by 10.13039/100012818Comunidad de Madrid and European Union under the FEDER program, from the European Union in response to COVID-19 pandemics. The funders had no role in the study design, data collection, analysis, preparation of the manuscript, or the decision to publish.

## Author contributions

L.H.M., D.H.A., G.S.F., N.C., V.I.D., and C.D. conducted research, provided experimental data, and contributed to analyzing data. L.H.M., J.M.B., and F.M. supervised research and drafted the manuscript. T.H. and F.M. supported the search for funding, planning the research, supervised the research, contributed to analyzing data, and wrote the manuscript.

## Declaration of interests

The authors declare that there are no conflicts of interest in connection with this article.
